# Risk of thrombotic events in immune thrombocytopenia patients treated with thrombopoietic agents: a systematic review and meta-analysis

**DOI:** 10.1186/s12959-023-00509-z

**Published:** 2023-06-23

**Authors:** Yu Dong, Zhinan Xia, Jie Zhou, Yutao Hu, Ming Yue, Yuyong Wang, Mengjiao Hu

**Affiliations:** 1 Department of the Fourth Clinical Medical College, Zhejiang Chinese Medical University, Hangzhou, China; 2 Department of Urology, The Fourth Affiliated Hospital, Zhejiang University School of Medicine, Yiwu, China; 3 Department of Urology, The First Affiliated Hospital, Harbin Medical University, Harbin, China; 4 Department of Basic Medicine College, Zhejiang Chinese Medical University, Hangzhou, China; 5 Department of the First Clinical Medical College, Zhejiang Chinese Medical University, Hangzhou, China; 6 Department of Urology, Affiliated Hangzhou First People’s Hospital, Zhejiang University School of Medicine, Hangzhou, China

**Keywords:** Immune thrombocytopenia, Platelet, Thrombopoietic agents, Thrombotic event, Meta-analysis

## Abstract

**Background:**

Immune thrombocytopenia (ITP), which is a well-known hemorrhagic disorder characterized by low platelet counts, has been shown to be associated with the risk of thrombosis. Thrombopoietic agents (TAs) are extensively used as second-line treatments for ITP, effectively reducing the risk of hemorrhage. However, thrombosis, a potential adverse effect of TAs, raises clinical challenges.

**Methods:**

The MEDLINE(PubMed), Embase, and the Cochrane Library databases were systematically searched for relevant studies, including both single-arm trials and randomized controlled trials (RCTs), without language restrictions.

**Results:**

A total of 17 RCTs comprising 2,105 patients and 29 single-arm trials comprising 3,227 patients were included. In the single-arm meta-analysis, the pooled rate of overall thrombotic events in ITP patients receiving TAs was 2.2% (95% CI 1.0% − 3.7%). In RCTs, a higher incidence of thrombosis (33/1425 vs. 4/680) and higher risk ratios (RR) of overall, arterial, and venous thrombotic events (1.73, 95% CI [0.88, 3.39], P = 0.113; RR 1.98, 95% CI [0.80, 4.92], P = 0.141; RR 1.06, 95% CI [0.46, 2.41], P = 0.895, respectively) were observed in the TAs group than in the control group, although the differences were not significant. Subgroup analysis demonstrated that hetrombopag was the only TA with no increased thrombotic risk (rate 0.3% 95% CI [0.0 − 1.5%]; RR 0.76, 95% CI [0.03, 18.41], P = 0.864) compared to eltrombopag, avatrombopag, romiplostim, and rhTPO. Subgroup analyses also revealed that ITP patients with advanced age (3.7% vs. 1.3%, P = 0.132) or with a thrombotic history (3.0% vs. 1.4%, P = 0.257), and patients who received TAs therapy for a long duration (4.7% vs. 0.1%, P < 0.001) had an increased risk of thrombosis.

**Conclusion:**

Our findings suggest ITP patients treated with TAs have a nonsignificantly higher risk of overall, arterial, and venous thrombotic events. Furthermore, hetrombopag is the recommended TA to avoid thrombophilia. Patients receiving long-term TAs, as well as elderly ITP patients or those with a history of thrombosis, face an increased thrombotic risk. In general, clinicians should consider potential thrombotic risks, address underlying risk factors, and ensure ongoing monitoring and follow-up when treating ITP patients with TAs.

**Supplementary Information:**

The online version contains supplementary material available at 10.1186/s12959-023-00509-z.

## Introduction

Primary immune thrombocytopenia (ITP) is a common acquired hemorrhagic disorder that can occur at any age, with an incidence of approximately 2–4 cases per 100,000 person-years [1]. The pathogenesis of ITP is heterogeneous, with the primary aetiologic mechanisms being the destruction of platelets and their precursors and the relative lack of platelet production, which leads to decreased peripheral platelet counts [2]. Thrombocytopenia can lead to bleeding symptoms, primarily manifesting in the skin and mucous membranes, and in severe cases, internal or even intracranial hemorrhage can occur. Platelet count thresholds associated with hemorrhage are < 20 × 10^9^ L^− 1^ or < 10 × 10^9^ L^− 1^ [3], and the risk of bleeding increases with age. Consequently, hematologists have long focused on bleeding events associated with low platelet counts in ITP patients, and treatment for ITP has been concentrated on reducing bleeding risk and increasing platelet counts.

However, a study conducted several years ago observed a high risk of thrombotic events in patients with ITP, which indicated that ITP patients might paradoxically be at risk for thrombosis [4]. Subsequent extensive epidemiological studies that matched ITP patients and non-ITP individuals by age and sex found that ITP patients have a higher risk of thrombosis [5–8]. Sarpatwari et al. analyzed 1,070 patients with chronic ITP and 4,280 ITP-free individuals, revealing that the adjusted hazard ratios for venous, arterial, and overall thrombosis in ITP patients compared to controls were 1.58 (1.01–2.48), 1.37 (0.94-2.00), and 1.41 (1.04–1.91), respectively [9]. Moreover, several systematic reviews [10, 11] have confirmed the increased arterial, venous, and overall thrombotic risks in patients with ITP, further suggesting that ITP is not only a hemorrhagic but also a thrombotic disorder. One possible explanation for the elevated thrombotic risk in ITP patients is their prothrombotic phenotype, which is characterized by preactivated platelets, activated endothelium, and elevated levels of coagulation factors [12–17]. These factors may collectively contribute to an increased risk of thrombosis in ITP patients, notwithstanding their low platelet counts.

Current ITP treatment is not strictly regimented. First-line therapy typically consists of steroids (high-dose dexamethasone or prednisone), intravenous immunoglobulin (IVIG), or even a combination of both for certain patients. Second-line treatment primarily includes rituximab, splenectomy, and thrombopoietic agents (TAs). TAs have become one of the common second-line treatments for ITP due to their great efficacy. TAs include first-generation recombinant human thrombopoietin (rhTPO) and second-generation thrombopoietin receptor agonists (TPO-RAs) [18]. TPO-RAs, including eltrombopag, romiplostim, hetrombopag, lusutrombopag, and avatrombopag, bear no similarity in structure with native thrombopoietin and therefore do not cross-react with the patients’ formed autoantibody. By mimicking TPO via c-Mpl binding, TAs activate JAK2/STAT5 signalling pathways, inducing megakaryocyte proliferation and differentiation, ultimately increasing circulating platelet count and reducing the risk of bleeding [19]. However, thrombotic adverse events of TAs in clinical practice have raised concerns among hematologists. A previous randomized controlled trial [20] reported that the incidence of thrombotic events was 2% for TAs compared with 0% for placebo. In a long-term open-label, single-arm study [21], the incidence of thrombotic events of TAs was as high as 8%.

The thrombotic risk of ITP patients and the contradiction between hemorrhage and thrombotic events during treatment with TAs have posed numerous clinical challenges. Additionally, rhTPO, the first-generation thrombopoietic agent, has been recommended by the Chinese guidelines for ITP treatment [22]. Several newly approved TPO-RAs, including avatrombopag and hetrombopag, have demonstrated therapeutic effectiveness [23, 24]. However, adverse events of those TAs, especially thrombotic events, remain uncertain. Therefore, to provide hematologists with a scientific basis for the clinical use of TAs, this meta-analysis was conducted to systematically evaluate the thrombotic risk of TAs in patients with ITP. Additionally, risk factors associated with thrombosis in ITP patients, and management options for thrombosis in ITP patients treated with TAs were also explored.

## Materials and methods

This meta-analysis was performed in accordance with the PRISMA (Preferred Reporting Items for Systematic Reviews and Meta-Analysis) statement [25]. The study protocol has been registered in the PROSPERO database (CRD42022346038).

### Search Strategy and Eligibility Criteria

We systemically searched the MEDLINE(PubMed), Embase, and the Cochrane Library databases with no language restrictions. Literature searches were conducted by using controlled vocabularies such as Medical Subject Headings (MeSH) or Emtree and free text words, including “Purpura, Thrombocytopenic, Idiopathic”, “ITP”, “autoimmune thrombocytopenia”, “immune thrombocytopenia”, “Autoimmune Thrombocytopenic Purpura”, “Immune Thrombocytopenic Purpura”, “thrombopoietic agents”, “recombinant human thrombopoietin”, “eltrombopag”, “romiplostim”, “avatrombopag”, “lusutrombopag”, “thrombopoietin receptor agonists”, “TPO”, “rhTPO”, “SB 497115 GR”, and “amg 531”. The search strategies were modified for each database and were presented in the supplemental data.

Studies were included if they met the following criteria:

1. Patient: adult patients with immune thrombocytopenia.
2. Intervention: use of thrombopoietic agents at any dosage, with or without combination therapy.
3. Comparison: standard-of-care or placebo.
4. Outcome: overall thrombotic events, arterial thrombotic events, and venous thrombotic events.
5. Study type: randomized controlled trial or single-arm trial.

Studies were excluded if they met the following criteria: (1) patients diagnosed with secondary immune thrombocytopenia; (2) duplicate publications; and (3) trials without available data.

### Data extraction and Quality Assessment

Three researchers (YD, YW, and MH) independently screened the titles and abstracts of the retrieved studies based on predefined eligibility criteria. Potentially relevant studies were further determined based on inclusion criteria by full-text screening. Data were then extracted from the included studies by three independent investigators (YD, YW, and MH) including (1) study characteristics (author, publication year, country in which the study was performed, funding source, study ID, study design, participant demographic); (2) baseline characteristics (age, sex, treatment duration, pretreatment platelet count, number of prior treatments, history of splenectomy); and (3) outcome events (number of patients who experienced overall thrombotic events, arterial thrombotic events, and venous thrombotic events). Discrepancies in study selection and data extraction were resolved through discussion.

The quality of the included single-arm trials was assessed by three independent reviewers (YD, YW, and MH) using the Methodological Index of Non-Randomized Studies (MINORS)[26]. The following items were assessed: (1) clear objectives of the trial; (2) inclusion of consecutive patients; (3) prospective data collection; (4) endpoints appropriate for the study objectives; (5) unbiased assessment of study endpoints; (6) follow-up period appropriate to the study objectives; (7) missed follow-up rate less than 5%, and (8) prospective calculation of study size. Items were scored as 0 (not reported), 1 (reported but inadequate), or 2 (reported and adequate). For single-arm studies, the ideal overall score was 16, with an overall score of more than 12 indicating high quality, scores from 8 to 12 indicating moderate quality, and scores less than 8 indicating poor quality. Disagreements about quality assessment were resolved by discussion.

The quality of the included randomized controlled trials was assessed by three independent reviewers (YD, YW, and MH) using the Cochrane risk of bias assessment instrument [27]. The following sources of bias were evaluated: random sequence generation, allocation concealment, blinding of participants and personnel, blinding of outcome assessment, incomplete outcome data, selective reporting, and other bias. Each item was graded as “low risk” or “high risk”; if there was insufficient information to judge, it was classified as “unclear”.

### Statistical analysis

For single-arm trials, descriptive statistics for the rate of patients with thrombotic events were calculated with a 95% CI and presented using forest plots. Due to the small number of events in some of the included trials and the presence of trials with no events, the Freeman-Tukey transformation was applied to conform the data to a normal distribution. Statistical heterogeneity was evaluated based on the I² test, with significant heterogeneity indicated if P > 0.10 and I² > 50%. Furthermore, random effects models were adopted for all meta-analyses due to the expected heterogeneity between trials. The pooled estimates obtained from the meta-analyses of Freeman-Tukey transformed proportions were then back-transformed, and the results were reported as rates. To investigate potential sources of heterogeneity, we conducted several subgroup analyses based on TAs subgroups, treatment duration (> 6 months or ≤ 6 months), whether patients with a history of thrombosis were excluded (yes or no), and patient age at baseline (> 50 years or ≤ 50 years). All data syntheses and statistical analyses were performed using Stata version 16 (College Station, TX).

For randomized controlled trials, all outcomes were dichotomous data calculated using risk ratios (RR) with a 95% confidence interval (CI). Statistical heterogeneity was determined based on the I² test, with significant heterogeneity indicated if P < 0.10 and I² > 50%. A fixed effect model was applied to estimate the pooled effect size if I² < 50% and P > 0.10; otherwise, a random effects model was adopted. Sensitivity analysis was conducted using a leave-one-out method to test the potential impact of each study on the pooled results and explore the robustness of the findings. Publication bias was assessed based on the symmetry of the funnel plot and the results of Egger’s and Begg’s tests.

## Results

### Study selection

**Fig. 1:**
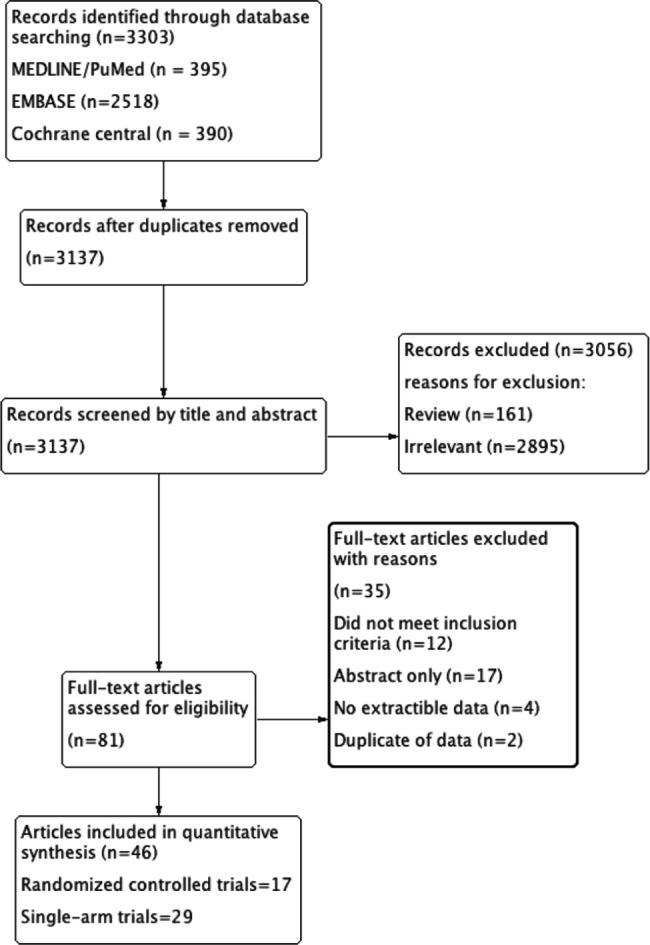
PRISMA flow diagram of the screening and selection process used in the study

A total of 3,303 records were identified through the initial database search. After removing duplicates, 3,137 records were screened by titles and abstracts, leaving 81 studies for full-text review. Four trials using lusutrombopag were excluded because they studied thrombocytopenia in patients with chronic liver disease, not primary immune thrombocytopenia patients, which did not meet our inclusion criteria. Ultimately, 46 studies (17 randomized controlled trials and 29 single-arm trials) that met the eligibility criteria were included in the meta-analysis (Fig. 1).

### Study characteristics

The study characteristics of the 29 single-arm trials [21, 28–54] are described in Table 1, and the patient baseline characteristics are shown in Table 2. All studies were published between 2006 and 2023, with sample sizes ranging from 10 to 407. A total of 3,227 adult patients were analyzed, 2,135 of whom were women, with the proportion of female ranging from 44% to 90%. Among the 29 trials investigating TAs, eleven studies investigated eltrombopag, two investigated hetrombopag, two investigated avatrombopag, thirteen investigated romiplostim, and one investigated rhTPO. Fourteen single-arm trials excluded patients with a thrombotic history.

**Table 1:** Characteristics of included single-arm trials

Study	Treatment duration	Therapy	Patients, n	Thrombotic events, n	History of thrombotic events as exclusion criteria (yes/no)
Tomiyama et al. 2012	6 months	Eltrombopag 12.5 mg	23	1: - Transient ischemic attack	Yes: excluded patients with a history of arterial or venous thrombosis within 1 year before enrolment
Brynes et al. 2017	116 weeks	Eltrombopag 50 mg, could be adjusted to 25 mg or 75 mg	118	4: - Pulmonary embolism - Deep vein thrombosis - Cerebral venous thrombosis - Transient ischemic attack	No
Bussel et al. 2013	6 weeks	Eltrombopag 50 mg, could be adjusted to 75 mg	66	0	Yes: excluded patients with history of thrombosis and two or more thrombophilic risk factors
G´omez-Almaguer et al. 2014	4 weeks	Eltrombopag 50 mg, Dexamethasone 40 mg	12	0	No
Haselboeck et al. 2013	4 weeks	Eltrombopag 25 mg, could be adjusted to 50 mg or 75 mg	10	2: - Deep vein thrombosis - Cerebral venous thrombosis	Yes: excluded patients with a history of thromboembolic disease
Kim et al. 2015	22 weeks	Eltrombopag 25 mg, could be adjusted to 12.5 mg	18	0	No
Liu et al. 2022	24 weeks	Eltrombopag 25 mg	150	0	Yes: excluded patients with any prior history of cardiovascular disease
Saleh et al. 2013	3 years	Eltrombopag 50 mg	299	20: 1 - Transient ischemic attack 1 - Central nervous ischemia 1 - Prolonged reversible ischemic neurologic deficit 1 - Subclavian/Brachial vein thrombosis 2 - Cerebral infraction 3 - Pulmonary embolism 3 - Myocardial infarction 8 - Deep vein thrombosis	Yes: excluded patients with a history of arterial or venous thrombosis, and two or more thrombophilic risk factors, or with any family history of arterial or venous thrombosis
Tripathi et al. 2014	4 weeks	Eltrombopag 50 mg	27	0	No
Wong et al. 2017	2 years	Eltrombopag 50 mg, could be adjusted to 25 mg or 75 mg	302	24: 1 - Thrombophlebitis superficial 1 - Pulmonary infarction 1 - Pulmonary embolism 1 - Cerebral ischemia 2 - Acute myocardial infarction 3 - Transient ischemic attack 3 - Myocardial infarction 4 - Cerebral infraction 8 - Deep vein thrombosis	Yes: excluded patients with a history of arterial or venous thrombosis, and two or more thrombophilic risk factors
van Dijk et al. 2023	Unknown	Eltrombopag 25 mg, could be adjusted to 50 mg or 75 mg	16	1: - Deep vein thrombosis	No
Mei et al. 2022	6 weeks	Hetrombopag 5 mg, could be adjusted to 2.5, 3.75, 5, 7.5 mg	37	0	Yes: excluded patients with arterial or venous thrombosis
Mei et al. 2021	24 weeks	Hetrombopag 2.5 or 5 mg, could be adjusted to 7.5 mg	275	2: - Acute myocardial infarction - Subclavian vein thrombosis	Yes: excluded patients with arterial or venous thrombosis
Bussel et al. 2014	24 weeks	Avatrombopag 10 mg, could be adjusted to 40 mg	53	2: - Iliac deep vein thrombosis - Myocardial infarction	Yes: excluded patients with history of cardiovascular disease, thromboembolic disease, deep vein thrombosis
Al-Samkari et al. 2022	90 weeks	Avatrombopag 20 mg	39	1: - Jugular vein thrombosis	Yes: excluded patients with clinically significant arterial or venous thrombosis and cardiovascular disease
Bussel et al. 2006	3 weeks	Romiplostim 0.2-1 µg/kg, or 3–10 µg/kg	24	0	Yes: excluded patients with any known risk factor for thromboembolic events or a history of cardiovascular disease
Gernsheimer et al. 2010	3 years	Romiplostim 1 or 2 µg/kg	101	8: 1 - Coronary artery occlusion 1 - Superficial vein thrombosis. 1 - Pulmonary embolism 1 - Septic jugular vein thrombosis 1 - Inflammatory venous thrombosis 1 - Transient cerebral ischemic attack 2 - Myocardial infarction	No
Bussel et al. 2009	144 weeks	Romiplostim 1–30 µg/kg	142	12 (in 7 patients, include: - Deep vein thrombosis - Myocardial infarction - Coronary artery occlusion - Septic thrombophlebitis - Transient ischemic attack)	No
Janssens et al. 2015	Median treatment duration was 44.3 (20.4, 65.9) weeks	Romiplostim 1 or 3 µg/kg	407	36: 1 - Device occlusion 1 - Ischemia stroke 1 - Thrombosis 1 - Myocardial infarction 1 - Hemiparesis 1 - Thrombophlebitis 1 - Splenic infarction 1 - Central venous catheterization 1 - Intestinal infarction 1 - Intracranial venous sinus thrombosis 2 - Transient ischemic attack 2 - Venous thrombosis 2 - Cerebrovascular accident 3 - Thrombosis in device 3 - Portal vein thrombosis 6 - Deep vein thrombosis 8 - Pulmonary embolism	No
Kuter et al. 2013	Up to 5 years	Romiplostim 1–30 µg/kg	292	25: 1 - Hemiparesis 1 - Transient blindness 1 - Transverse sinus thrombosis 1 - Portal vein thrombosis 1 - Thrombophlebitis 1 - Catheter thrombosis 2 - Transient ischemic attack 2 - Cerebrovascular accident 2 - Pulmonary embolism 3 - Deep vein thrombosis 10 - Myocardial infarction	No
Mihaylov et al. 2020	24 months	Romiplostim 1 µg/kg, could be adjusted to 4.5 µg/kg	100	1: - Thrombosis	No
Newland et al. 2006	3 weeks	Romiplostim 30, 100, 300, 500 µg	16	0	Yes: excluded patients with a history of arterial or untreated venous thrombotic disease, and three or more thromboembolic risk factors
Newland et al. 2015	12 months	Romiplostim 1–10 µg/kg	75	1: - Reversible ischaemic neurological deficit	Yes: excluded patients with history of recurrent venous thromboembolism or thrombotic events within 5 years of enrolment
Park et al. 2016	24 weeks	Romiplostim 1 µg/kg	18	0	No
Reiser et al. 2021	Up to 2 years	Romiplostim 1–10 µg/kg	96	0	No
Shirasugi et al. 2012	13 weeks	Romiplostim 3–10 µg/kg	44	1: - Transient ischemic attack	No
Singh et al. 2022	8 weeks	Romiplostim 1–5 µg/kg	50	1: - Mild lacunar infarct	No
Steurer et al. 2016	2 years	Romiplostim, median dose was 2.8 µg/kg	340	10: 1 - embolism 1 - myocardial infarction 1 - retinal vein thrombosis 1 - Transient ischemic attack 1 - Thrombosed haemorrhoids 1 - Thrombophlebitis 2 - Deep vein thrombosis 2 - Pulmonary embolism	No
Cai et al. 2017	14 weeks	rhTPO, 300 U/kg	77	0	Yes: excluded patients with history of thrombotic disease

**Table 2:** Baseline characteristics of the patients from single-arm trials

Study	Females, n (%)	Age, years, Mean (SD) or Median (range)	Platelet counts per 10^9^/L (SD or range)	Previous splenectomy, n (%)	Number of previous treatments, n (%)
Tomiyama et al. 2012	15 (65)	60 (26–72)	17 (10–24)	16 (70)	19 (83)
Brynes et al. 2017	104 (64)	42 (18–80)	59 (36)^a^	37 (23)	0 (0)
Bussel et al. 2013	45 (68)	51 (20–79)	4 (6)^b^	20 (30)	≥ 3: 29 (44)
G´omez-Almaguer et al. 2014	6 (50)	50 (20–80)	7 (2–28)	NA	NA
Haselboeck et al. 2013	9 (90)	30 (20–58)	NA	0 (0)	Median number of treatments: 2 (1–3)
Kim et al. 2015	11 (61)	55 (30–71)	14 (1–28)	4 (22)	Median number of treatments: 3 (2–9)
Liu et al. 2022	112 (75)	44 (15)	20 (15)	25 (17)	≥ 1: 76 (51)
Saleh et al. 2013	198 (66)	50 (18–86)	128 (43)^a^	115 (38)	≥ 3: 47 (16)
Tripathi et al. 2014	15 (60)	27 (9)	14 (5)	0 (0)	0 (0)
Wong et al. 2017	201 (67)	49 (16)	211 (70)^c^	115 (38)	≥ 3: 160 (53)
van Dijk et al. 2023	7 (44)	53 (14)	23 (9–27)	2 (12)	NA
Mei et al. 2022	25 (68)	40 (28–53)	14 (11–22)	3 (8)	≥ 1: 37 (100)
Mei et al. 2021	241 (88)	41 (18–74)	13 (1–29)	29 (11)	NA
Bussel et al. 2014	38 (72)	50 (18)	15 (28)^a^	17 (32)	NA
Al-Samkari et al. 2022	23 (59)	46 (14)	18 (46)^a^	11 (28)	≥ 1: 15 (38)
Bussel et al. 2006	17 (71)	45 (21–65)	9 (4–31)	19 (79)	1–3: 9 (38) 4–6: 12 (50) > 6: 3 (13)
Gernsheimer et al. 2010	81 (80)	52 (21–88)	16 (2–31)	83 (82)	≥ 3: 79 (78)
Bussel et al. 2009	96 (67)	53 (21–89)	17 (1–50)	86 (60)	≥ 1: 32 (22)
Janssens et al. 2015	244 (60)	56 (18–93)	14 (0–170)	208 (51)	≥ 1: 208 (51)
Kuter et al. 2013	184 (63)	54 (17)	35 (15–100)	95 (33)	≥ 1: 37 (13)
Mihaylov et al. 2020	56 (56)	45 (27–58)	19 (8–42)	23 (23)	≥ 3: 49 (49)
Newland et al. 2006	10 (63)	50 (20–84)	15 (6–31)	13 (81)	NA
Newland et al. 2015	44 (59)	39 (29–57)	20 (12–25)	0 (0)	≥ 1: 44 (57)
Park et al. 2016	12 (67)	40.5 (26–73)	14 (4–30)	4 (22)	≥ 3: 8 (44)
Reiser et al. 2021	50 (48)	67 (55–72)	29 (15–78)	9 (9)	≥ 1: 85 (89)
Shirasugi et al. 2012	31 (71)	56 (25–81)	17 (3–32)	17 (39)	NA
Singh et al. 2022	33 (66)	36 (12)	NA	NA	≥ 1: 50 (100)
Steurer et al. 2016	183 (54)	62 (46–72)	20 (9–35)	116 (34)	≥ 3: 186 (55)
Cai et al. 2017	44 (57)	37 (3–74)	10 (0–44)	NA	NA

NA: not available

^a^ Platelet counts ≤ 15 × 10^9^/L, n (%)

^b^ Platelet counts ≤ 20 × 10^9^/L, n (%)

^c^ Platelet counts ≤ 30 × 10^9^/L, n (%)

The study characteristics of the 17 RCTs [20, 29, 43, 45, 53, 55–66] are described in Table 3, and the patient baseline characteristics are shown in Table 4. All studies were published between 2006 and 2021, with sample sizes ranging from 21 to 424. A total of 2,105 adult patients were analysed, 1,410 of whom were women, with the proportion of female ranging from 47% to 88%. Among the 17 trials, six studies investigated eltrombopag, one investigated hetrombopag, two investigated avatrombopag, four investigated romiplostim, and four investigated rhTPO. Fourteen studies excluded patients with a history of thrombosis.

**Table 3:** Characteristics of included randomized controlled trials

Study	Treatment duration	TAs		Control		Thrombotic events, n		History of thrombotic events as exclusion criteria, yes/no
		Therapy	Patients, n	Method	Patients, n	TAs group	Control group	
Bussel et al. 2007	6 weeks	Eltrombopag 30 mg, 50 mg, or 75 mg	88	Placebo	29	1: - Thromboembolism in the small vessels of the liver and kidneys	0	Yes: excluded patients with thrombosis within 1 year before enrolment or myocardial infarction within 3 months before enrolment
Bussel et al. 2009	6 weeks	Eltrombopag 50 or 75 mg	76	Placebo	38	0	0	Yes: excluded patients with thrombosis within the previous years
Cheng et al. 2011	24 weeks	Eltrombopag 50 mg, could be adjusted to 25 mg or 75 mg	135	Placebo	62	3: 1 - Deep vein thrombosis 2 - Pulmonary embolism	0	Yes: excluded patients with arterial or venous thrombosis plus two or more thrombosis risk factors
Huang et al. 2018	6 weeks	Eltrombopag 25 mg, could be adjusted to 25 mg or 75 mg	17	Placebo	18	1: - Cerebral infarction	0	Yes: excluded patients with history of arterial/venous thrombosis plus two or more thrombotic risk factors.
Tomiyama et al. 2012	6 weeks	Eltrombopag 12.5–25 mg	15	Placebo	8	1: - Transient ischemic attack	0	Yes: excluded patients with a history of arterial or venous thrombosis within 1 year before enrolment
Yang et al. 2016	8 weeks	Eltrombopag 25–75 mg	104	Placebo	51	2: - Cerebral infarction - Deep vein thrombosis	0	Yes: excluded patients with any prior history of cardiovascular disease
Mei et al. 2021	10 weeks	Hetrombopag 2.5 or 5 mg	339	Placebo	85	1: - Acute myocardial infarction	0	Yes: excluded patients with venous or arterial thrombosis
Bussel et al. 2014	4 weeks	Avatrombopag 2.5, 5, 10, 20 mg	59	Placebo	5	5: - Stroke - Myocardial infarction - Retinal artery occlusion - Iliac deep vein thrombosis - Superficial thrombophlebitis	0	Yes: excluded patients with history of cardiovascular disease, thromboembolic disease, deep vein thrombosis
Jurczak et al. 2018	26 weeks	Avatrombopag 20 mg, could be adjusted to 40 mg or 5 mg	32	Placebo	17	3: - Deep vein thrombosis - Asymptomatic pulmonary embolism - Cerebrovascular event	0	Yes: excluded patients with clinically significant arterial or venous thrombosis and cardiovascular disease
Bussel et al. 2006	6 weeks	Romiplostim 1, 3 or 6 µg/kg	17	Placebo	4	0	1: - Popliteal deep vein thrombosis	Yes: any known risk factor for thromboembolic events or a history of cardiovascular disease
Kuter et al. 2008	24 weeks	Romiplostim 1 or 2 µg/kg	83	Placebo	42	2: - Popliteal artery thrombosis. - Stroke	1: - Pulmonary embolism	No
Kuter et al. 2010	52 weeks	Romiplostim 3–10 µg/kg	157	Standard of care	77	11 (in 6 patients, include: 1 - Myocardial infarction 2 - Deep vein thrombosis 3 - Pulmonary embolism)	2 (in 2 patients)	No
Shirasugi et al. 2011	12 weeks	Romiplostim 3–10 µg/kg	22	Placebo	12	0	0	Yes: excluded patients with arterial thrombosis or a history of venous thrombosis necessitating anticoagulation therapy
Gu et al. 2013	1 week	rhTPO 15000U, Methyllprednisolone 80 mg	31	Methyllprednisolone 80 mg	31	1: - Acute myocardial infarction	0	Yes: excluded patients with history of thrombotic events
Wang et al. 2012	4 weeks	rhTPO 1 µg/kg, Danazol 200 mg	73	Danazol 200 mg	67	0	0	Yes: excluded patients with history of thrombosis
Yu et al. 2020	2 weeks	rhTPO 300 U/kg, Dexamethasone 40 mg	100	Dexamethasone 40 mg	96	1: - Cerebral infarction	0	Yes: excluded patients with a history of arterial or venous thrombosis
Zhou et al. 2015	2 weeks	rhTPO 300 U/kg, Rituximab 100 mg	77	Rituximab 100 mg	38	1: - Myocardial infarction	0	No

**Table 4:** Baseline characteristics of the patients from randomized controlled trials

Study	Females, n (%)		Age, years, Mean (SD) or Median (range)		Platelet counts per 10^9^/L Mean (SD) or Median (range)		Previous splenectomy, n (%)		Number of previous treatments, n (%)	
	TAs group	Control group	TAs group	Control group	TAs group	Control group	TAs group	Control group	TAs group	Control group
Bussel et al. 2007	73 (62)	16 (55)	50 (18–85)	42 (18–85)	42 (48)^a^	14 (48)^a^	41 (47)	14 (48)	≥ 3: 60 (51)	≥ 3: 14 (48)
Bussel et al. 2009	43 (57)	27 (71)	47 (19–84)	51 (21–79)	38 (50)^a^	17 (45)^a^	31 (41)	14 (37)	≥ 3: 42 (55)	≥ 3: 16 (42)
Cheng et al. 2011	93 (69)	43 (69)	47 (34–56)	52 (43–63)	16 (8–22)	16 (9–24)	50 (37)	21 (34)	≥ 3: 75 (56)	≥ 3: 32 (52)
Huang et al. 2018	15 (88)	14 (78)	50 (24–62)	40 (22–66)	14 (4–27)	14 (1–26)	NA	NA	≥ 1: 9 (53)	≥ 1: 7 (39)
Tomiyama et al. 2012	8 (53)	7 (88)	58 (26–72)	61 (38–72)	21 (16–25)	10 (8–19)	11 (73)	5 (63)	NA	NA
Yang et al. 2016	77 (74)	40 (78)	48 (18–84)	42 (22–66)	54 (51.9) ^a^	28 (55) ^a^	18 (17)	7 (14)	≥ 1: 19 (18)	≥ 1: 10 (20)
Mei et al. 2021	241 (71)	60 (71)	41 (18–74)	42 (18–71)	13 (1–29)	13 (1–29)	29 (9)	4 (5)	NA	NA
Bussel et al. 2014	37 (63)	3 (60)	54 (18)	40 (21)	16 (27)	2 (40)	18 (31)	2 (40)	NA	NA
Jurczak et al. 2018	23 (72)	8 (47)	46 (14)	41 (15)	18 (56) ^a^	10 (59) ^a^	11 (34)	5 (29)	≥ 1: 15 (47)	≥ 1: 7 (41)
Bussel et al. 2006	12 (71)	3 (75)	45 (19–63)	55 (39–64)	15 (4–25)	29 (6–49)	13 (76)	1 (25)	1–3: 5 (29) 4–6: 9 (53) > 6: 3 (18)	1–3: 1(25) 4–6: 3 (75) > 6: 0 (0)
Kuter et al. 2008	54 (65)	27 (64)	52 (21–88)	52 (23–88)	16 (2–29)	18 (2–31)	42 (51)	21 (50)	≥ 3: 54 (65)	≥ 3: 26 (60)
Kuter et al. 2010	85 (54)	46 (60)	58 (18–90)	57 (18–86)	33 (1–123)	27 (2–62)	0 (0)	0 (0)	≥ 2: 110 (70)	≥ 2: 60 (78)
Shirasugi et al. 2011	14 (64)	10 (83)	59 (13)	28 (13)	18 (8)	16 (1)	10 (46)	5 (42)	Median number of treatments: 4 (1–19)	Median number of treatments: 4 (1–7)
Gu et al. 2013	20 (65)	18 (58)	52 (22–80)	48 (21–84)	7 (1–10)	7 (1–10)	0 (0)	0 (0)	NA	NA
Wang et al. 2012	53 (73)	33 (52)	41 (18–74)	41(18–74)	11 (2–20)	10 (1–20)	9 (12)	8 (13)	NA	NA
Yu et al. 2020	67 (67)	65 (68)	42 (19–74)	45 (22–73)	7 (0–24)	7 (0–30)	2 (2)	1 (1)	≥ 1: 37 (37)	≥ 1: 44 (46)
Zhou et al. 2015	50 (65)	25 (66)	42 (13–82)	42.5 (12–68)	9 (0–30)	13 (2–30)	9 (12)	3 (8)	≥ 1: 43 (57)	≥ 1: 19 (50)

TAs: Thrombopoietic agents

NA: not available

^a^ Platelet counts ≤ 15 × 10^9^/L, n (%)

### Quality Assessment

The 29 included single-arm studies were assessed by the MINORS; the scores ranged from 9 to 14, with a mean score of 12.21 ± 0.846. Nine single-arm trials scored greater than 12 and were determined to be of high quality, and the remaining 20 trials had scores between 8 and 12 and were therefore assessed as moderate quality (Supplemental Table 1).

**Fig. 2:**
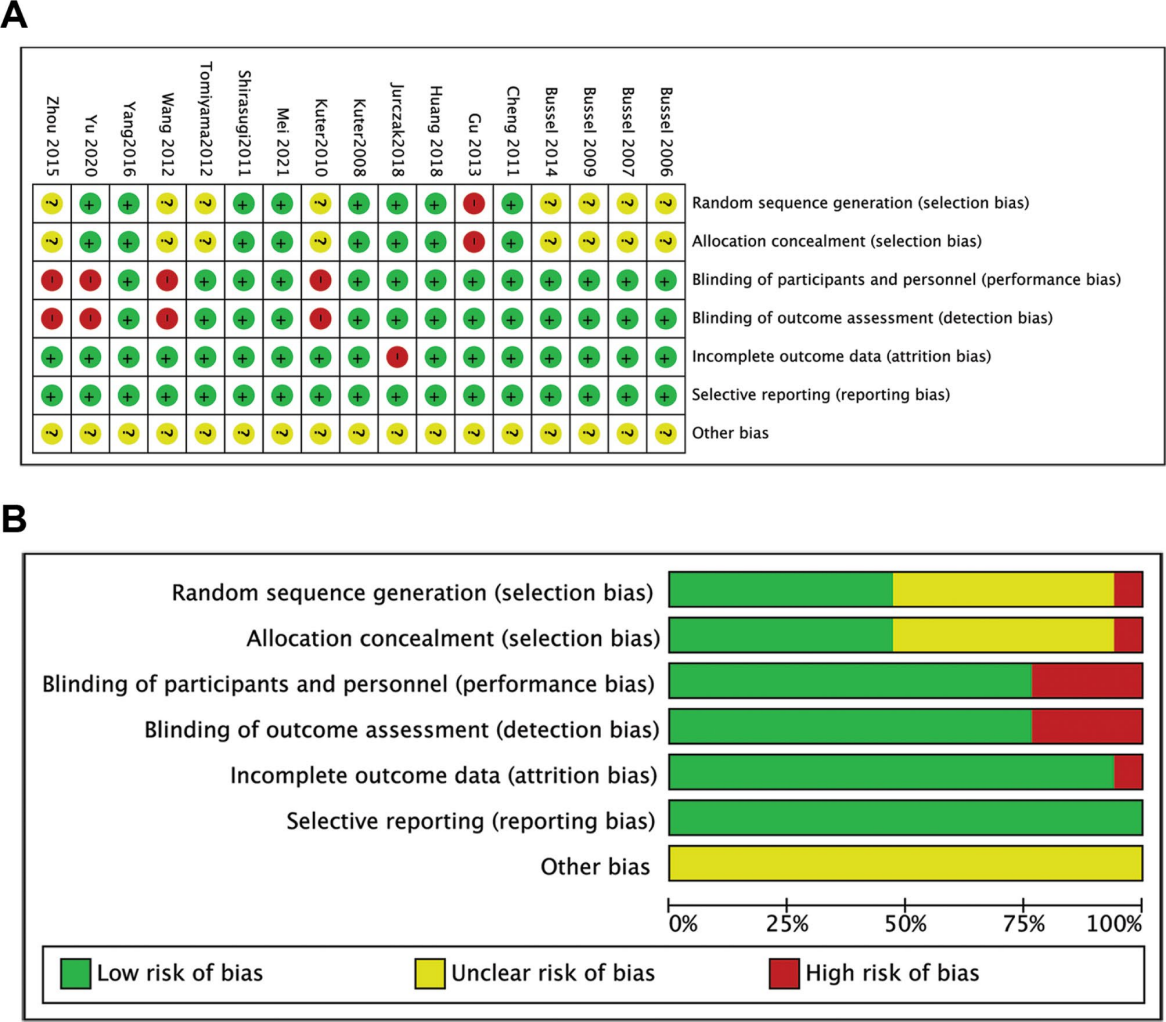
Summary (A) and graph (B) of the risk of bias in the included randomized controlled trials by the Cochrane risk of the bias assessment instrument. Assessments were based on the reviewers’ judgment of each domain

Among randomized controlled trials, one study [55] was assessed to have a high selection risk due to the random generation of sequences based on the order of patient enrolment. Four studies [56–58, 60] were open-label trials and, therefore, were considered to have a high risk of performance bias. One study [62] was considered to have a high risk of attrition bias as 16 of 17 patients who received placebo treatment discontinued the study. Overall, all included trials were considered to have a low risk of bias as evaluated by the Cochrane risk of bias assessment instrument (Fig. 2).

### Primary outcome

#### Overall thrombotic events

**Fig. 3:**
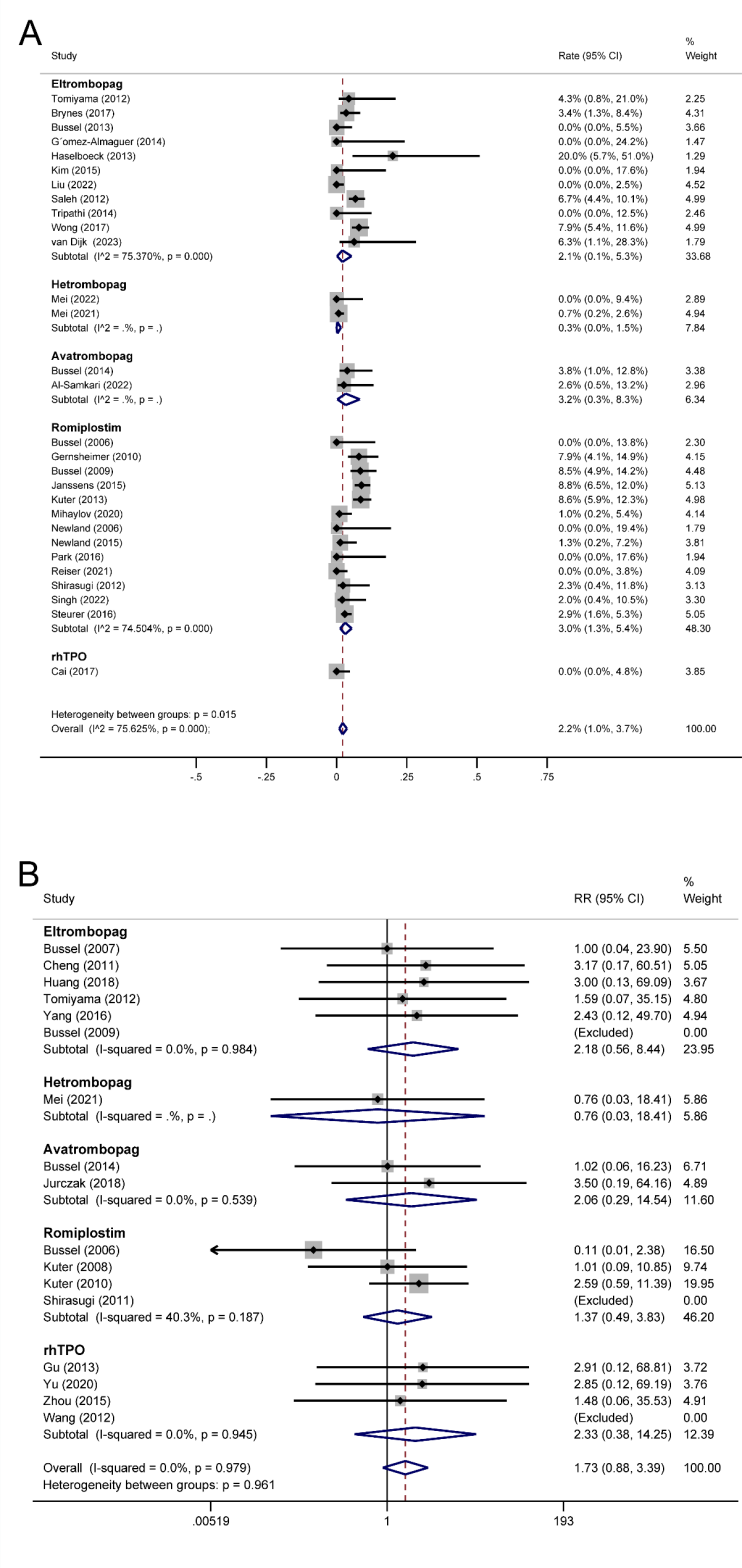
Forest plot of overall thrombotic events in ITP patients treated with TAs. (A) Forest plot of rate for overall thrombotic events after ITP patients treated with TAs in single-arm trials. (B) Forest plot of RR for overall thrombotic events after ITP patients treated with TAs in randomized controlled trials. RR: risk ratio. ITP: immune thrombocytopenia. TAs: thrombopoietic agents. CI: confidence interval

A total of 29 single-arm trials reporting overall thrombotic events during the TAs treatment period were included in the meta-analysis (n = 3,227). The pooled rate of overall thrombotic events in TAs using a random effect model was 2.2% (95% CI 1.0% − 3.7%) (Fig. 3A). The pooled thrombotic rates were 2.1% (95% CI 0.1% − 5.3%) for eltrombopag, 0.3% (95% CI 0.0% − 1.5%) for hetrombopag, 3.2% (95% CI 0.3% − 8.3%) for avatrombopag, 3.0% (95% CI 1.3% − 5.4%) for romiplostim (Fig. 3A).

In single-arm meta-analyses, significant heterogeneity was observed in the pooled rates of overall thrombotic events for TAs treatment (I^2^ = 75.625%, P < 0.001), eltrombopag treatment (I^2^ = 75.370%, P < 0.001), and romiplostim treatment (I^2^ = 74.504%, P < 0.001). Subgroup analysis was performed based on treatment duration, patient age at baseline, and whether patients with a thrombotic history were excluded. These indicators failed to eliminate the heterogeneity of TAs treatment (Table 5) and romiplostim treatment (Supplemental Table 2), indicating that other potential sources of heterogeneity might exist. For the eltrombopag treatment subgroup, the significant heterogeneity was eliminated by the indicator of treatment duration (I^2^ = 46.1%, P = 0.084 vs. I^2^ = 32.053%, P = 0.23, Supplemental Table 2), suggesting that the difference in treatment duration across studies might be a source of heterogeneity. Furthermore, the thrombotic event rates of the eltrombopag subgroup were higher in studies with longer treatment durations than in studies with shorter treatment durations (6.4%, 95% CI [4.3 − 8.8%] vs. 0.1%, 95% CI [0.0 − 2.6%], P = 0. 009).

**Table 5:** Subgroup analyses for pooled rates of overall thrombotic events in single-arm trials

Study	No. of studies	Rate (95% CI)	Heterogeneity		*p* for subgroup difference
			*I*^2^ (%)	*p*	
**Thrombopoietic agents**					
Overall	29	2.2% (1.0 − 3.7%)	75.625	< 0. 001	NA
**Treatment duration**					< 0. 001
≤ 6 months	16	0.1% (0.0 − 0.7%)	11.109	0.326	
> 6 months	12	4.7% (2.9 − 6.8%)	76.437	< 0.001	
**Excluded patients with thrombotic history**					0.257
Yes	14	1.4% (0.1 − 3.7%)	77.771	< 0.001	
No	15	3.0% (1.3 − 5.2%)	69.816	< 0.001	
**Age, years**					0.132
≤ 50	18	1.3% (0.2 − 3.0%)	70.149	< 0.001	
> 50	11	3.7% (1.6 − 6.6%)	77.145	< 0.001	

CI: confidence interval. NA: not available. Since the treatment duration data for van Dijk et al. 2023 was unavailable, this trial was not included in the subgroup analysis based on treatment duration

Subgroup analysis demonstrated that the incidence of overall thrombotic events was significantly higher in patients receiving TAs for more than 6 months than in those treated with TAs for less than 6 months (4.7%, 95% CI [2.9 − 6.8%] vs. 0.1%, 95% CI [0.0 − 0.7%], P < 0. 001, Table 5). The incidence of thrombosis was higher in studies that did not exclude patients with a history of thrombosis than in those that did (3.0%, 95% CI [1.3 − 5.2%] vs. 1.4%, 95% CI [0.1 − 3.7%], P = 0. 257). Patients older than 50 years were found to have a higher risk of overall thrombotic events than those younger than 50 years (3.7%, 95% CI [1.6 − 6.6%] vs. 1.3%, 95% CI [0.2 − 3.0%], P = 0. 132).

The overall thrombotic events in patients treated with TAs were retrieved from 17 randomized controlled trials (n = 2,105). More thrombotic events occurred in the TAs group than in the control group: 33/1425 versus 4/680, respectively. Out of the 14 RCTs that could be used to estimate the RR for overall thrombotic events, 12 showed an RR of 1 or higher. According to a meta-analysis using a fixed effects model, patients treated with TAs were more likely to experience thrombosis than patients who received standard-of-care or placebo (RR 1.73, 95% CI [0.88, 3.39], P = 0.113, Fig. 3B), while the difference was not statistically significant. Moreover, subgroup analysis indicated that, although not statistically significant, eltrombopag (RR 2.18, 95% CI [0.56, 8.44], P = 0.261), avatrombopag (RR 2.06, 95% CI [0.29, 14.54], P = 0.468), romiplostim (RR 1.37, 95% CI [0.49, 3.83], P = 0.548), and rhTPO (RR 2.33, 95% CI [0.38, 14.25], P = 0.361) treatments were associated with an increased thrombotic risk among TAs therapies, while hetrombopag was not (RR 0.76, 95% CI [0.03, 18.41], P = 0.864, Fig. 3B). We further conducted pairwise subgroup analysis between hetrombopag and other subgroups of TAs. Compared to other TAs, hetrombopag showed a significantly lower incidence of thrombotic events and was the only drug with a risk ratio smaller than 1 when compared to the control group, indicating that hetrombopag was indeed the TA with the lowest risk of thrombosis (Supplemental Table 3).

### Secondary outcomes

#### Arterial thrombotic events

**Fig. 4:**
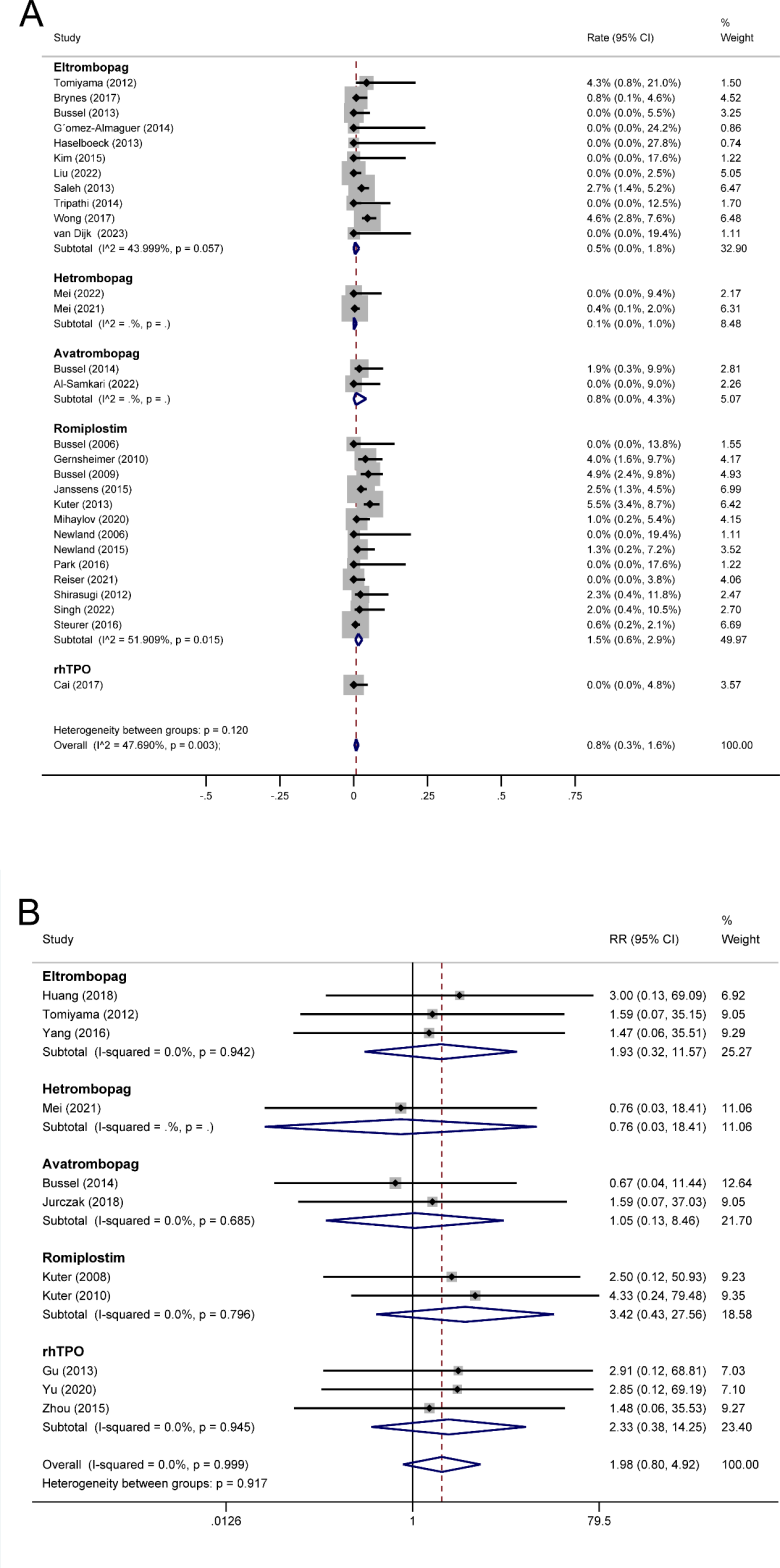
Forest plot of arterial thrombotic events in ITP patients treated with TAs. (A) Forest plot of rate for arterial thrombotic events after ITP patients treated with TAs in single-arm trials. (B) Forest plot of RR for arterial thrombotic events after ITP patients treated with TAs in randomized controlled trials. RR: risk ratio. ITP: immune thrombocytopenia. TAs: thrombopoietic agents. CI: confidence interval

A total of 3,227 patients from 29 single-arm studies were assessed for the incidence of arterial thrombotic events that occurred during the treatment period of TAs. According to a pooled analysis using a random effects model, the pooled rate of arterial thrombotic events for TAs was 0.8% (95% CI 0.3% − 1.6%), the rate for eltrombopag was 0.5% (95% CI 0.0% − 1.8%), the rate for hetrombopag was 0.1% (95% CI 0.0% − 1.0%), the rate for avatrombopag was 0.8% (95% CI 0.0% − 4.3%), and the rate for romiplostim was 1.5% (95% CI 0.6% − 2.9%) (Fig. 4A).

Among the 17 randomized controlled trials of TAs treatment included in the meta-analysis, 11 reported arterial thrombotic events. The TAs group experienced more arterial thrombotic events than the control group: 17/1014 versus 0/468, respectively. A fixed-effects model meta-analysis revealed that the TAs group had a higher risk of arterial thrombotic events than the control group (RR 1.98, 95% CI [0.80, 4.92], P = 0.141, Fig. 4B), but the difference was not statistically significant. Moreover, except for hetrombopag (RR 0.76, 95% CI [0.03, 18.41], P = 0.864), other TAs such as eltrombopag (RR 1.93, 95% CI [0.32, 11.57], P = 0.471), avatrombopag (RR 1.05, 95% CI [0.13, 8.46], P = 0.963), romiplostim (RR 3.42, 95% CI [0.43, 27.56], P = 0.248) and rhTPO (RR 2.33, 95% CI [0.38, 14.25], P = 0.361), were associated with increased risks of arterial thrombotic events (Fig. 4B), though the results were not significantly different across subgroups. Based on the frequency and clinical significance of different types of events in arterial thrombosis, we classified arterial thrombosis types and then conducted meta-analyses, which revealed that myocardial ischemia and cerebral ischemia may be the most common arterial thrombotic events (Supplemental Fig. 1, Supplemental Fig. 2, Supplemental Fig. 3, and Supplemental Fig. 4).

### Venous thrombotic events

**Fig. 5:**
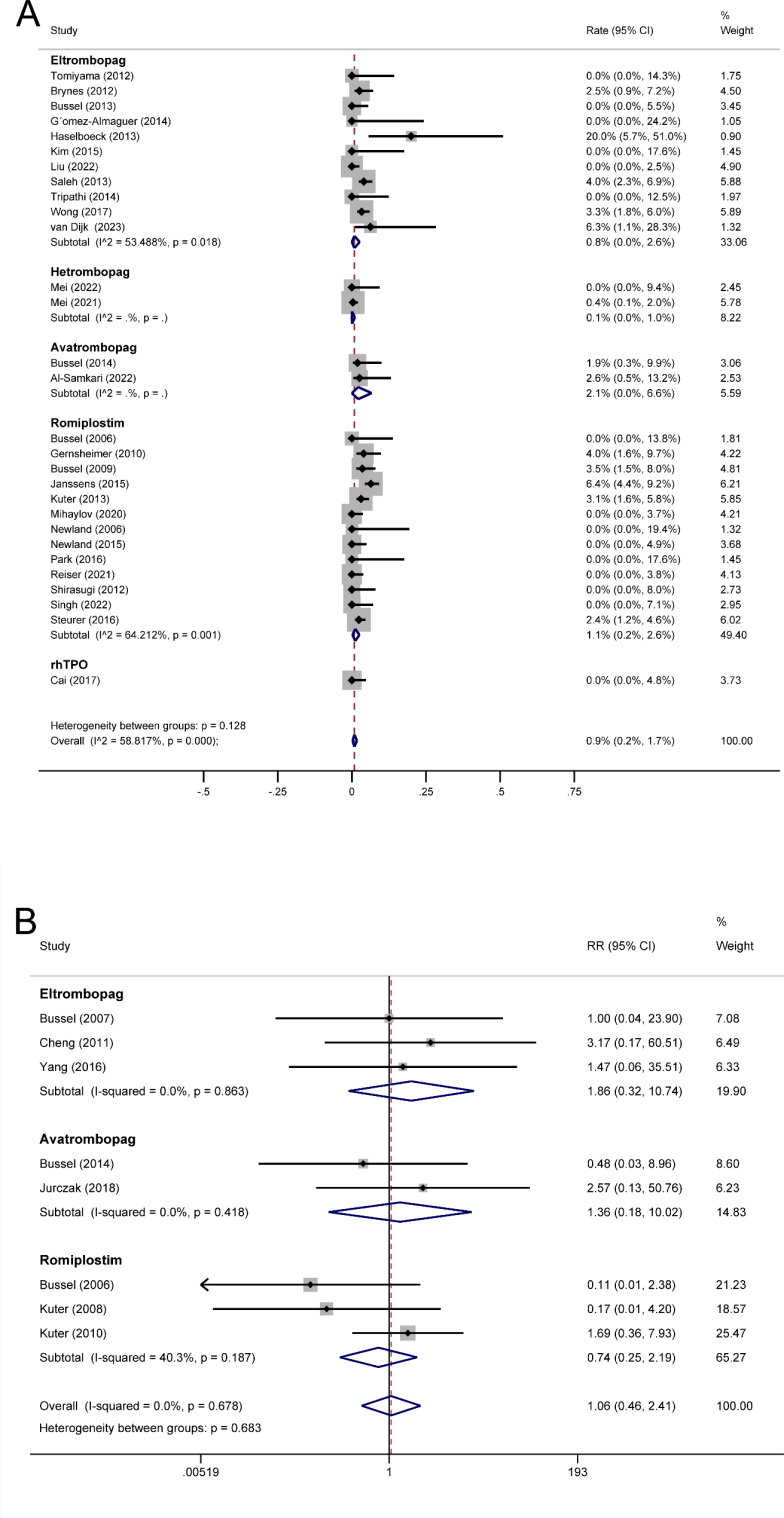
Forest plot of venous thrombotic events in ITP patients treated with TAs. (A) Forest plot of rate for venous thrombotic events after ITP patients treated with TAs in single-arm trials. (B) Forest plot of RR for venous thrombotic events after ITP patients treated with TAs in randomized controlled trials. RR: risk ratio. ITP: immune thrombocytopenia. TAs: thrombopoietic agents. CI: confidence interval

A total of 29 single-arm trials reporting venous thrombotic events that occurred during the treatment period of TAs were included in the meta-analysis (n = 3,227). Using a random effects model, the pooled rate of venous thrombotic events in TAs was 0.9% (95% CI 0.2% − 1.7%) (Fig. 5A), whereas the pooled rates for eltrombopag were 0.8% (95% CI 0.0% − 2.6%), for hetrombopag were 0.1% (95% CI 0.0% − 1.0%), for avatrombopag were 2.1% (95% CI 0.0% − 6.6%), and for romiplostim were 1.1% (95% CI 0.2% − 2.6%) (Fig. 5A).

Eight randomized controlled trials (n = 962) reported venous thrombotic events in ITP patients who received TAs therapy. The incidence of venous thrombotic events was 16/675 in the TAs group versus 4/287 in the control group. The fixed effect model revealed that TAs were associated with an increased risk of venous thrombotic events (RR 1.06, 95% CI [0.46, 2.41], P = 0.895, Fig. 5B), although the difference was not significant. Subgroup analysis suggested that eltrombopag and avatrombopag might be associated with an increased risk of venous thrombosis (RR 1.86, 95% CI [0.32, 10.74], P = 0.488 and RR 1.36, 95% CI [0.18, 10.02], P = 0.623), while romiplostim did not show such an association (RR 0.74, 95% CI [0.25, 2.19], P = 0.591). The results for each TAs subgroup were not statistically significant. We classified venous thrombosis types based on the frequency and clinical significance of various arterial thrombotic events. Subsequently, we conducted meta-analyses and determined that deep vein thrombosis was likely the most prevalent venous thrombotic event (Supplemental Fig. 5, Supplemental Fig. 6, Supplemental Fig. 7, and Supplemental Fig. 8).

### Sensitivity analyses and publication bias

Sensitivity analyses were performed using the leave-one-out method and did not identify any individual study that significantly changed pooled RRs, this indicating the stability of the results of meta-analyses for overall, arterial, and venous thrombotic events among randomized controlled trials (Supplemental Fig. 9, Supplemental Fig. 10, and Supplemental Fig. 11). The symmetry of the funnel plot suggested no evidence of publication bias (Supplemental Fig. 12). The results of Egger’s and Begg’s tests indicated that there was no evidence of potential publication bias in randomized controlled trials of overall thrombotic events (P = 0.447 and P = 0.381), arterial thrombotic events (P = 0.735 and P = 0.35), and venous thromboembolic events (P = 0.57 and P = 0.536).

## Discussion

TAs have demonstrated great efficacy in increasing platelet counts > 50 × 10^9^/L in 60-90% of adults with ITP and have been widely applied as second-line therapy in ITP patients [67]. Thrombosis, which is as a potential adverse event associated with TAs, may pose significant challenges to clinical management, while its exact incidence remains unknown. Our meta-analysis examined classic rhTPO, romiplostim, and eltrombopag as well as novel TAs such as hetrombopag and avatrombopag to systematically explore the thrombotic rate and risk of TAs.

Our analysis of single-arm studies demonstrated that the overall thrombotic event rate in TA-treated ITP patients was 2.1%, with arterial and venous thrombosis occurring in 0.9% and 0.8% of cases, respectively. ITP patients inherently possess a risk of thrombosis. To further differentiate the extrinsic thrombosis risk factors for TAs therapy from disease-intrinsic risk factors, we then included randomized controlled trials in our meta-analysis. Our findings indicated that TAs treatment was associated with a higher risk of overall, arterial, and venous thrombotic events in ITP patients (RR = 1.73, RR = 1.98, and RR = 1.06). Among the 17 trials reporting overall thrombotic events, a higher rate of events occurred in the TAs group than in the control group (33/1425 vs. 4/680). Out of the 14 randomized controlled trials that could be used to evaluate the RR of overall thrombotic events, 12 trials showed an RR equal to or greater than 1, indicating a higher risk of thrombotic events in the TAs group than in the control group. However, it appears that the increased number of thrombotic events does not translate into a significant difference.

The subgroup analysis of single-arm and randomized controlled trials revealed that in TAs, both first-generation rhTPO and second-generation drugs, such as romiplostim, eltrombopag, and avatrombopag, were associated with an increased incidence of overall thrombotic events. In contrast, hetrombopag did not demonstrate such an association. Additionally, the subgroup differences between hetrombopag and the classical TPO-RAs, eltrombopag and romiplostim, was statistically significant (P = 0.038 and P = 0.007, respectively), suggesting that hetrombopag might be a preferable choice when there is suspicion of thrombosis in patients requiring treatment with TAs.

Exploring risk factors for thrombosis in patients receiving TAs therapy has significant clinical implications, as it can guide clinicians in avoiding the challenging situation of thrombosis when treating patients with similar risk factors. Higher rates of thrombotic events have been associated with intrinsic risk factors (age, sex, previous thrombotic history, comorbidities, and antiphospholipid antibodies) and extrinsic risk factors. Our subgroup analysis indicated that long-term use of TAs was linked to a higher risk of thrombosis, suggesting that treatment exposure duration might be a risk factor for thrombosis. For example, a 5-year open-label study by Kuter et al. reported that the prevalence of tthrombosis among those undergoing romiplostim treatment was 6.5%. It should be noted that, as maintenance therapy, TAs are often long-term or even life-long continuous therapy [67]. Therefore, when prescribing TAs, it is crucial to ensure continuous monitoring of thrombosis risk. Our subgroup analysis indicated that TAs should be administered cautiously in older patients, as advanced age is a factor contributing to an increased incidence of thrombosis. This result was in line with previous studies that found older age to be an independent risk factor for thrombosis in ITP patients [68–71]. Our subgroup analysis also showed that the incidence of thrombosis was higher in studies that included patients with a history of thrombosis, suggesting that a history of thrombosis might be an independent thrombotic risk factor for ITP patients receiving TAs therapy. This outcome was consistent with previous research [9, 68, 69]. The relationship between sex and thrombotic risk remains controversial. Two population-based studies suggested that the male sex was associated with a higher risk of arterial thrombosis [68, 69], while another study found that sex was not a risk factor [72]. Comorbidities, such as obesity, hypertension, and diabetes, have been shown to increase the risk of thrombosis, as well as cardiovascular disease, and the latter might increase the risk of arterial thrombosis, especially myocardial infarction [9, 70, 72]. As an independent risk factor for thrombosis in ITP patients, antiphospholipid antibody (APL) is frequently present in hospitalized patients with ITP, with a reported prevalence of 25–75%[73]. A 5-year follow-up study of ITP patients found that APL-positive patients had significantly lower cumulative thrombosis-free survival (39% vs. 97.7%, P = 0.0004) [73], indicating that APL could be an independent risk factor for thrombosis.

For patients with additional thrombotic risk factors, it is essential to address and modify individual risk factors and implement thrombosis prophylaxis [74]. When treating ITP patients, clinicians should be aware of the thrombotic risk and maintain long-term follow-up after administrating TAs. Managing and balancing bleeding and thrombosis can be challenging once thrombosis develops in ITP patients treated with TAs. Currently, there are no specific guidelines for managing thrombosis in patients with ITP, nor are there any guidelines for the platelet count threshold of antithrombotic therapy. When dealing with patients who have thrombocytopenia and thrombosis, a personalized approach is necessary. If the risk of hemorrhage is life-threatening, prioritizing treatments such as hemostasis and platelet elevation is essential. Once platelets reach safe levels, carefully assessing the patient’s status allows for the administration of appropriate microcirculation-improving therapy to prevent thrombosis. In cases where the risk of thrombosis is life-threatening, antiplatelet and anticoagulation treatments should be favoured. The platelet threshold is often used as a criterion for evaluating the safety of antithrombotic therapy in ITP patients. In 2016, a study by Samuelson et al. recommended a platelet count threshold of 50 × 10^9^/L for therapeutic anticoagulation in patients with venous thromboembolism and chemotherapy-induced thrombocytopenia [75]. In 2018, Pishko et al. summarized the perspectives of ITP specialists and hematologists-oncologists on the minimum platelet count for antiplatelet or anticoagulant therapy. The survey revealed that, despite opinions varying, the most recommended platelet count threshold for antiplatelet or anticoagulant therapy in ITP patients with thrombotic events was 50 × 10^9^/L [76]. Al-Samkari, on the other hand, argued that anticoagulant therapy should continue in ITP patients unless the disease is refractory to all treatments and a minimum platelet count (e.g., ≥ 20 × 10^9^/L) cannot be achieved [77]. The platelet safety threshold for anticoagulation and antiplatelet therapy requires further investigation and evidence-based research. In general, the management of thrombosis in ITP patients should be individualized, taking into account thrombotic risk factors, bleeding risk, and the severity of thrombotic events.

### Comparison to the literature

A previous systematic review conducted by Tjepkema et al. studied the risk of thrombosis with TPO-RAs, which found TPO-RAs were linked to a higher but nonsignificant risk of thrombosis in ITP patients. Our study explored a similar research topic to that of Tjepkema et al. but employed a different research design. First, we aim to study not only the overall thrombotic risk of the TPO-RAs class but also the specific risks of each drug within the TAs class, such as rhTPO, eltrombopag, hetrombopag, avatrombopag and romiplostim, to provide more specific information. Second, the treatment of arterial and venous thrombosis differs clinically, so we also investigated the risks of each subtype, not just the overall thrombotic risk. Third, we included not only RCTs but also single-arm studies in our literature search to provide more comprehensive information, including both risk ratios and event rates. Fourth, we further explored the risk factors for thrombosis through subgroup analysis.

Apart from differences in study design, there were variations in analysis approaches. Our search strategy was more comprehensive, allowing us to identify additional RCTs [43, 45, 66] that were not included in Tjepkema et al.‘s review. Moreover, we excluded a study [49] from the RCTs meta-analysis, and instead treated its experimental group as a single-arm study to ensure more reliable analysis results. This decision was made because the study was a nonrandomized controlled trial, and combining it with RCTs was inappropriate.

### Limitations

There were several limitations of our meta-analysis. First most included studies did not report thrombotic events as primary outcome indicators but rather as adverse events. Consequently, the lack of detailed information on thrombotic events hindered any comprehensive analyses, such as the evaluation of risk factors. Additionally, there were several variations in study designs and TAs dosages among the included trials. Moreover, the short follow-up time in several studies might render thrombotic events not fully documented. Furthermore, the small sample sizes of several included studies limited our ability to distinguish potentially subtle differences.

## Conclusion

In summary, the findings of this systematic review and meta-analysis indicated that there are nonsignificantly higher chances of overall, arterial, and venous thrombotic events in ITP patients treated with TAs. Hetrombopag was recommended as a TA that did not demonstrate a propensity for thrombophilia. In addition, patients receiving long-term TAs treatment and elderly patients or patients with a history of thrombosis were more susceptible to experiencing thrombotic events. When treating ITP patients with TAs, it is imperative to contemplate the possible thrombotic risks, address contributing risk factors, and ensure continuous monitoring and follow-up. Once thrombosis has occurred, a detailed evaluation and individualized treatment are needed.

## Electronic supplementary material

Below is the link to the electronic supplementary material.

Supplementary Material 1
