# Supplementary material for: Risk of thrombotic events in immune thrombocytopenia patients treated with thrombopoietic agents: a systematic review and meta-analysis
Source: Thromb J. 2023 Jun 23;21:69. doi: 10.1186/s12959-023-00509-z (PMC10290365; doi:10.1186/s12959-023-00509-z)
Supplement: Supplementary file 1 — Supplementary Material 1 [file 12959_2023_509_MOESM1_ESM.docx]

**Supplementary material**

**Supplemental File 1: Search strategy**

#1: (((((Purpura, Thrombocytopenic, Idiopathic[mesh]) OR (immune thrombocytopenia[Title/Abstract])) OR (ITP[Title/Abstract])) OR (autoimmune thrombocytopenia[Title/Abstract])) OR (Immune Thrombocytopenic Purpura[Title/Abstract])) OR (Autoimmune Thrombocytopenic Purpura[Title/Abstract])

#2: ((((((((((thrombopoietic agents[Title/Abstract]) OR (recombinant human thrombopoietin[Title/Abstract])) OR (eltrombopag[Title/Abstract])) OR (romiplostim[Title/Abstract])) OR (avatrombopag[Title/Abstract])) OR (lusutrombopag[Title/Abstract])) OR (thrombopoietin receptor agonists[Title/Abstract])) OR (TPO[Title/Abstract])) OR (rhtpo[Title/Abstract])) OR (SB 497115 GR[Title/Abstract])) OR (amg 531[Title/Abstract])

#3: #1 and #2

**Supplemental Table 1. Quality assessment of single-arm trials by using MINORS.**

| **Study** | **①** | **②** | **③** | **④** | **⑤** | **⑥** | **⑦** | **⑧** | **Total score** |
| --- | --- | --- | --- | --- | --- | --- | --- | --- | --- |
| Tomiyama et al. 2012 | 2 | 2 | 2 | 2 | 1 | 2 | 2 | 0 | 13 |
| Brynes et al. 2017 | 2 | 2 | 2 | 2 | 1 | 2 | 1 | 0 | 12 |
| Bussel et al. 2013 | 2 | 2 | 2 | 2 | 1 | 1 | 1 | 0 | 12 |
| G´omez-Almaguer et al. 2014 | 2 | 2 | 2 | 2 | 1 | 2 | 2 | 0 | 13 |
| Haselboeck et al. 2013 | 2 | 2 | 2 | 2 | 1 | 1 | 2 | 0 | 12 |
| Kim et al. 2015 | 2 | 2 | 2 | 2 | 0 | 2 | 2 | 0 | 12 |
| Liu et al. 2022 | 2 | 2 | 2 | 2 | 1 | 2 | 1 | 0 | 12 |
| Saleh et al. 2013 | 2 | 2 | 2 | 2 | 1 | 2 | 1 | 0 | 12 |
| Tripathi et al. 2014 | 2 | 2 | 2 | 2 | 0 | 1 | 0 | 0 | 9 |
| Wong et al. 2017 | 2 | 2 | 2 | 2 | 1 | 2 | 1 | 0 | 12 |
| van Dijk et al. 2023 | 2 | 2 | 2 | 2 | 1 | 2 | 1 | 2 | 14 |
| Mei et al. 2022 | 2 | 2 | 2 | 2 | 1 | 1 | 2 | 0 | 12 |
| Mei et al. 2021 | 2 | 2 | 2 | 2 | 1 | 2 | 1 | 0 | 12 |
| Bussel et al. 2014 | 2 | 2 | 2 | 2 | 1 | 2 | 1 | 0 | 12 |
| Al-Samkari et al. 2022 | 2 | 2 | 2 | 2 | 0 | 2 | 2 | 0 | 12 |
| Bussel et al. 2006 | 2 | 2 | 2 | 2 | 1 | 1 | 2 | 0 | 12 |
| Gernsheimer et al. 2010 | 2 | 2 | 2 | 2 | 1 | 2 | 1 | 0 | 12 |
| Bussel et al. 2009 | 2 | 2 | 2 | 2 | 1 | 2 | 1 | 0 | 12 |
| Janssens et al. 2015 | 2 | 2 | 2 | 2 | 1 | 2 | 2 | 0 | 13 |
| Kuter et al. 2013 | 2 | 2 | 2 | 2 | 1 | 2 | 2 | 0 | 13 |
| Mihaylov et al. 2020 | 2 | 2 | 2 | 2 | 0 | 2 | 1 | 0 | 11 |
| Newland et al. 2006 | 2 | 2 | 2 | 2 | 1 | 1 | 2 | 0 | 12 |
| Newland et al. 2015 | 2 | 2 | 2 | 2 | 1 | 2 | 2 | 0 | 13 |
| Park et al. 2016 | 2 | 2 | 2 | 2 | 1 | 2 | 2 | 0 | 13 |
| Reiser et al. 2021 | 2 | 2 | 2 | 2 | 1 | 2 | 2 | 0 | 13 |
| Shirasugi et al. 2012 | 2 | 2 | 2 | 2 | 1 | 1 | 2 | 0 | 12 |
| Singh et al. 2022 | 2 | 2 | 2 | 2 | 1 | 1 | 2 | 0 | 12 |
| Steurer et al. 2016 | 2 | 2 | 2 | 2 | 1 | 2 | 2 | 0 | 13 |
| Cai et al. 2017 | 2 | 2 | 2 | 2 | 1 | 1 | 2 | 0 | 12 |

①: A clearly stated aim. ②: Inclusion of consecutive patients. ③: Prospective collection of data. ④: Endpoints appropriate to the aim of the study. ⑤: Unbiased assessment of the study endpoint. ⑥: Follow-up period appropriate to the aim of the study. ⑦: Loss to follow up less than 5%. ⑧: Prospective calculation of the study size. The items are scored 0 (not reported), 1 (reported but inadequate) or 2 (reported and adequate). The global ideal score being 16 for single-arm studies.

**Supplemental Table 2. Subgroup analyses for pooled rates of overall thrombotic events of eltrombopag and romiplostim in single-arm trials.**

| **Study** | **No. of studies** | **Rate**  **(95% CI)** | **Heterogeneity** | | ***p* for subgroup difference** | **Study** | **No. of studies** | **Rate**  **(95% CI)** | **Heterogeneity** | | ***p* for subgroup difference** |
| --- | --- | --- | --- | --- | --- | --- | --- | --- | --- | --- | --- |
|  |  |  | ***I*^2^ (%)** | ***p*** |  |  |  |  | ***I*^2^ (%)** | ***p*** |  |
| **Eltrombopag** |  |  |  |  |  | **Romiplostim** |  |  |  |  |  |
| Overall | 11 | 2.1%  (0.1% - 5.3%) | 75.370 | < 0.001 | NA | Overall | 13 | 3.0%  (1.3% - 5.4%) | 74.504 | < 0.001 | NA |
| Treatment duration |  |  |  |  | 0.009 | Treatment duration |  |  |  |  | 0.207 |
| ≤ 6 months | 7 | 0.1%  (0.0% - 2.6%) | 46.100 | 0.084 |  | ≤ 6 months | 5 | 0.7%  (0.0% - 3.5%) | 0 | 0.960 |  |
| > 6 months | 3 | 6.4%  (4.3% - 8.8%) | 32.053 | 0.230 |  | > 6 months | 8 | 4.3%  (1.9% - 7.3%) | 83.370 | < 0.001 |  |
| Excluded patients with thrombotic history |  |  |  |  | 0.675 | Excluded patients with thrombotic history |  |  |  |  | 0.125 |
| Yes | 6 | 3.0%  (0.1% - 8.4%) | 86.603 | < 0.001 |  | Yes | 3 | 0.3%  (0.0% - 3.1%) | 0 | 0.946 |  |
| No | 5 | 1.3%  (0.0% - 4.1%) | 0 | 0.699 |  | No | 10 | 3.8%  (1.7% - 6.6%) | 78.444 | < 0.001 |  |
| Age, years |  |  |  |  | 0.518 | Age, years |  |  |  |  | 0.021 |
| ≤ 50 | 7 | 2.6%  (0.1% - 7.0%) | 81.724 | < 0.001 |  | ≤ 50 | 6 | 0.5%  (0.0% - 2.2%) | 0 | 0.993 |  |
| > 50 | 4 | 0.8%  (0.0% - 5.3%) | 32.495 | 0.217 |  | > 50 | 7 | 5.1%  (2.5% - 8.4%) | 81.974 | < 0.001 |  |

CI: confidence interval. Since the treatment duration data for van Dijk et al. 2023 was unavailable, this trial was not included in the subgroup analysis based on treatment duration.

| **Thrombopoietic agents** | **Single-arm**  **Meta-analysis** | | **RCT**  **Meta-analysis** | |
| --- | --- | --- | --- | --- |
|  | **Rate**  **(95% CI)** | ***p* for subgroup difference** | ***RR***  **(95% CI)** | ***p* for subgroup difference** |
| Hetrombopag | 0.3%  (0.0% - 1.5%) | NA | 0.76  (0.03 - 18.41) | NA |
| Eltrombopag | 2.1%  (0.1% - 5.3%) | 0.038 | 2.18  (0.56 - 8.44) | 0.608 |
| Avatrombopag | 3.2%  (0.3% - 8.3%) | 0.057 | 2.06  (0.29 - 14.54) | 0.601 |
| Romiplostim | 3.0%  (1.3% - 5.4%) | 0.007 | 1.37  (0.49 - 3.83) | 0.756 |
| rhTPO | 0.0%  (0.0% - 4.8%) | 0.565 | 2.33  (0.38 - 14.25) | 0.604 |

**Supplemental Table 3. Pairwise subgroup analysis of heterombopag compared to other subgroups.**

NA: not available


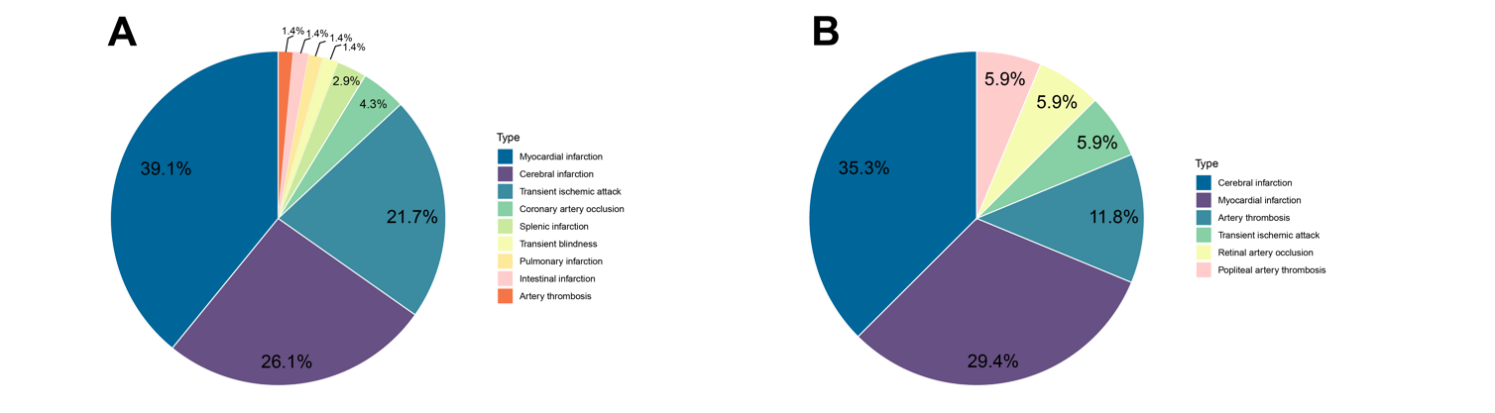


Supplemental Figure 1. Distribution of arterial thrombosis subtypes in single-arm trials and randomized controlled trials. (A) Distribution of arterial thrombosis subtypes in single-arm trials. (B) Distribution of arterial thrombosis subtypes in randomized controlled trials.


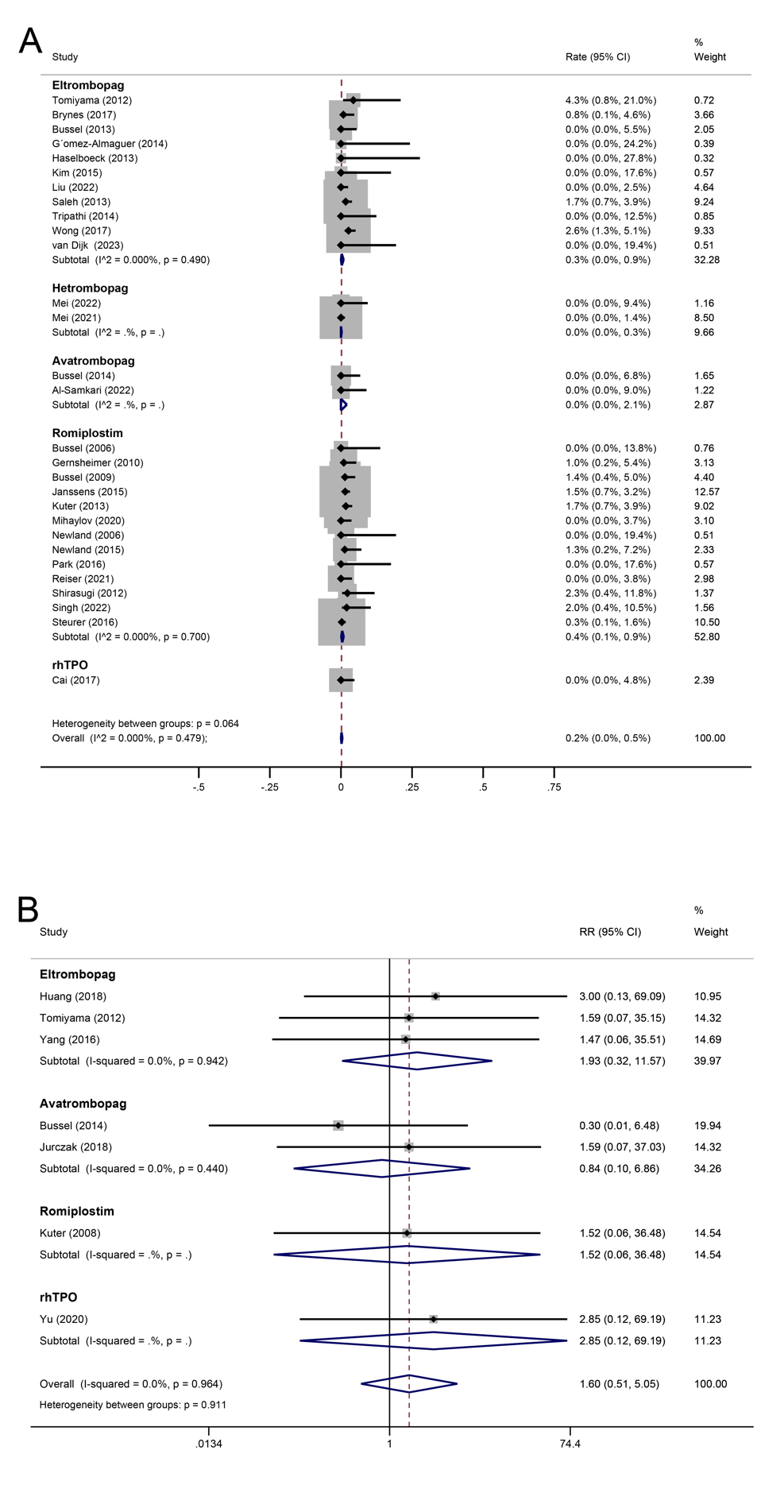


Supplemental Figure 2. Forest plot of cerebral ischemia events in ITP patients treated with TAs. (A) Forest plot of rate for cerebral ischemia events after ITP patients treated with TAs in single-arm trials. (B) Forest plot of RR for cerebral ischemia events after ITP patients treated with TAs in randomized controlled trials. RR: risk ratio. ITP: immune thrombocytopenia. TAs: thrombopoietic agents. CI: confidence interval. Cerebral ischemia events: including cerebral infarction and transient ischemic attack.


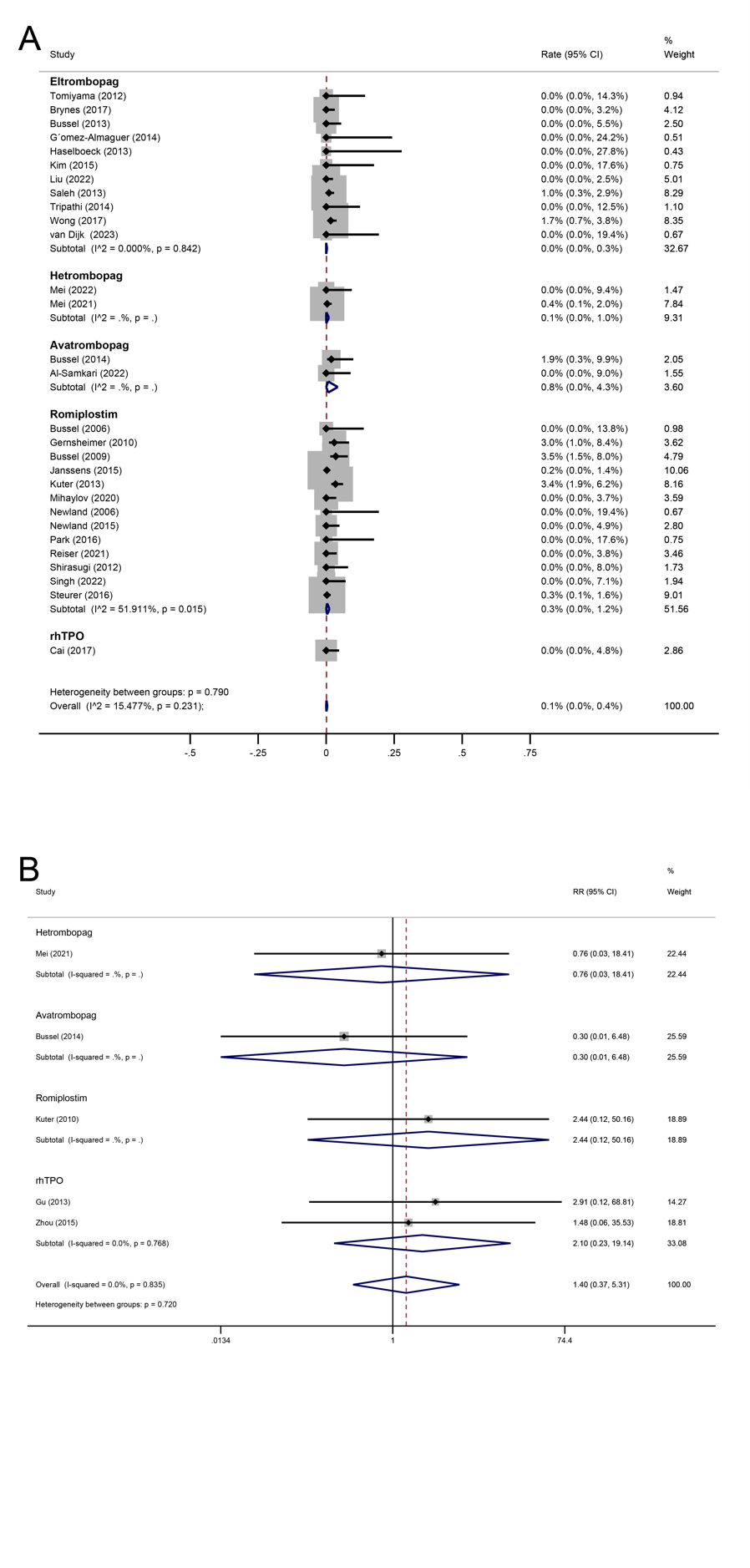


Supplemental Figure 3. Forest plot of myocardial ischemia events in ITP patients treated with TAs. (A) Forest plot of rate for myocardial ischemia events after ITP patients treated with TAs in single-arm trials. (B) Forest plot of RR for myocardial ischemia events after ITP patients treated with TAs in randomized controlled trials. RR: risk ratio. ITP: immune thrombocytopenia. TAs: thrombopoietic agents. CI: confidence interval. Myocardial ischemia events: including myocardial infarction and coronary artery occlusion.


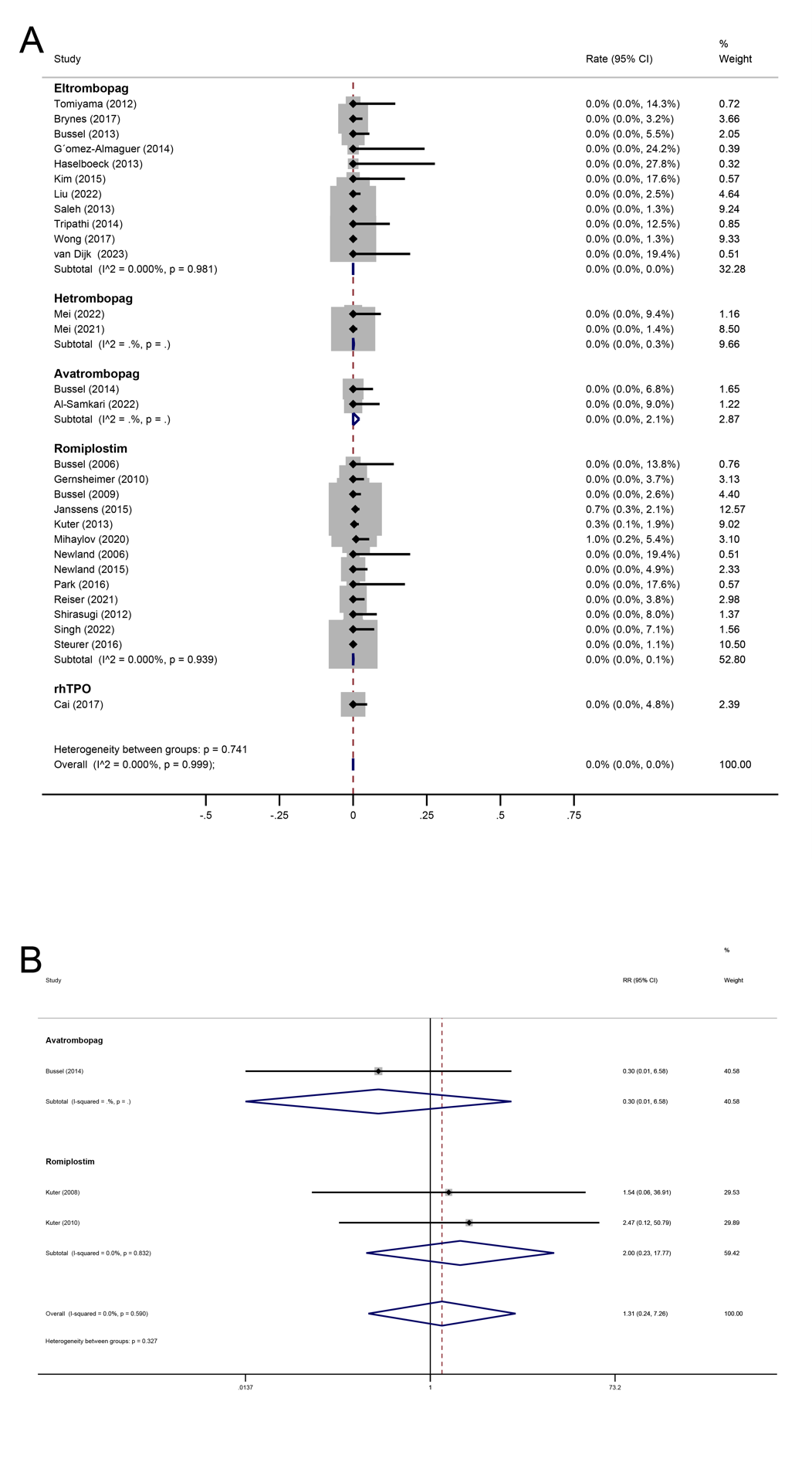


Supplemental Figure 4. Forest plot of other types of arterial thrombosis in ITP patients treated with TAs. (A) Forest plot of rate for other types of arterial thrombosis after ITP patients treated with TAs in single-arm trials. (B) Forest plot of RR for other types of arterial thrombosis after ITP patients treated with TAs in randomized controlled trials. RR: risk ratio. ITP: immune thrombocytopenia. TAs: thrombopoietic agents. CI: confidence interval.


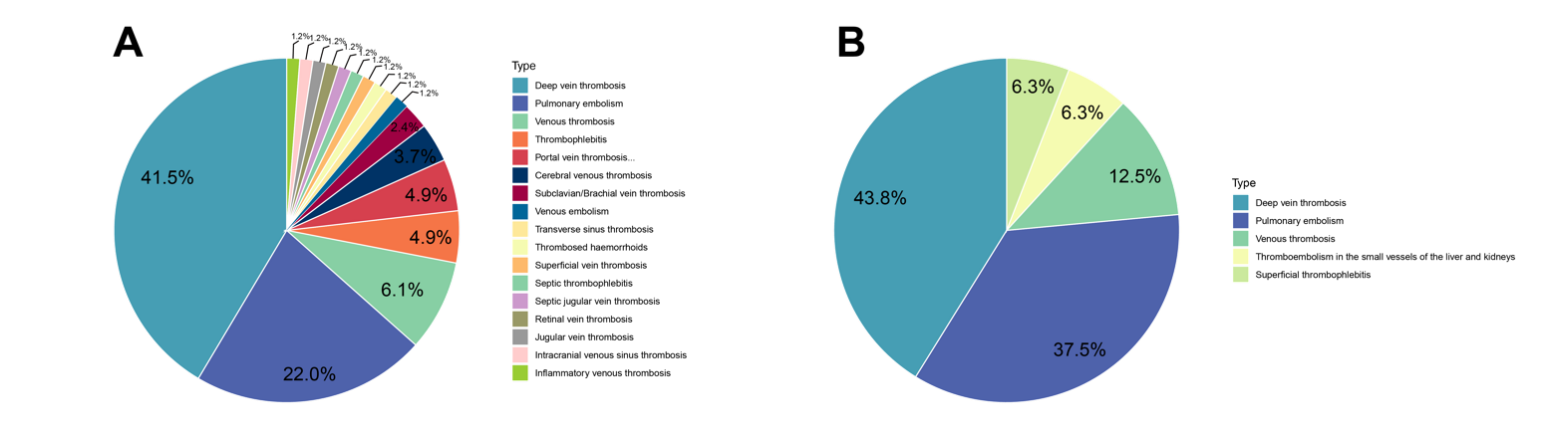


Supplemental Figure 5. Distribution of venous thrombosis subtypes in single-arm trials and randomized controlled trials. (A) Distribution of venous thrombosis subtypes in single-arm trials. (B) Distribution of venous thrombosis subtypes in randomized controlled trials.


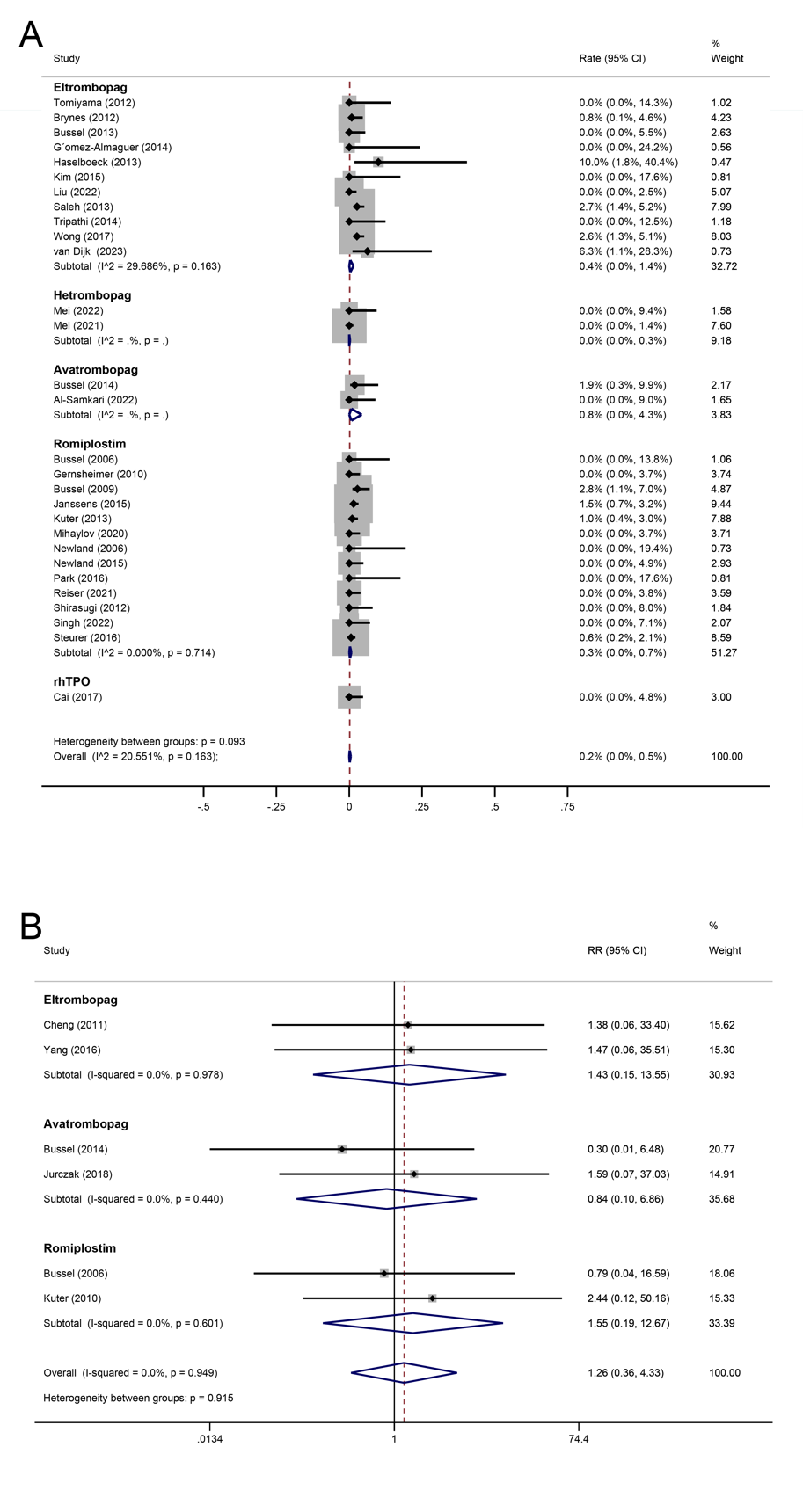


Supplemental Figure 6. Forest plot of deep vein thrombosis in ITP patients treated with TAs. (A) Forest plot of rate for deep vein thrombosis after ITP patients treated with TAs in single-arm trials. (B) Forest plot of RR for deep vein thrombosis after ITP patients treated with TAs in randomized controlled trials. RR: risk ratio. ITP: immune thrombocytopenia. TAs: thrombopoietic agents. CI: confidence interval.


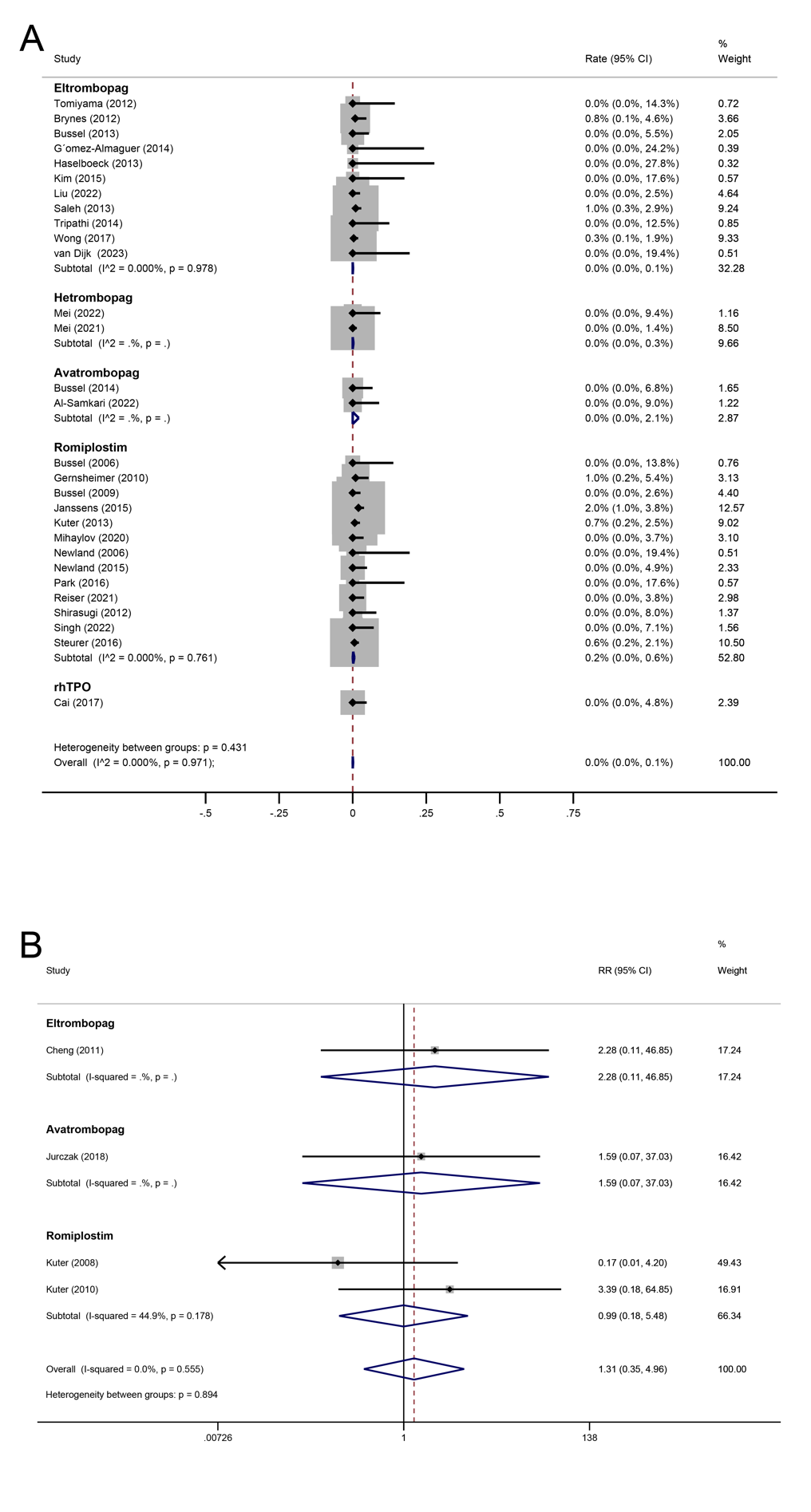


Supplemental Figure 7. Forest plot of pulmonary embolism in ITP patients treated with TAs. (A) Forest plot of rate for pulmonary embolism after ITP patients treated with TAs in single-arm trials. (B) Forest plot of RR for pulmonary embolism after ITP patients treated with TAs in randomized controlled trials. RR: risk ratio. ITP: immune thrombocytopenia. TAs: thrombopoietic agents. CI: confidence interval.


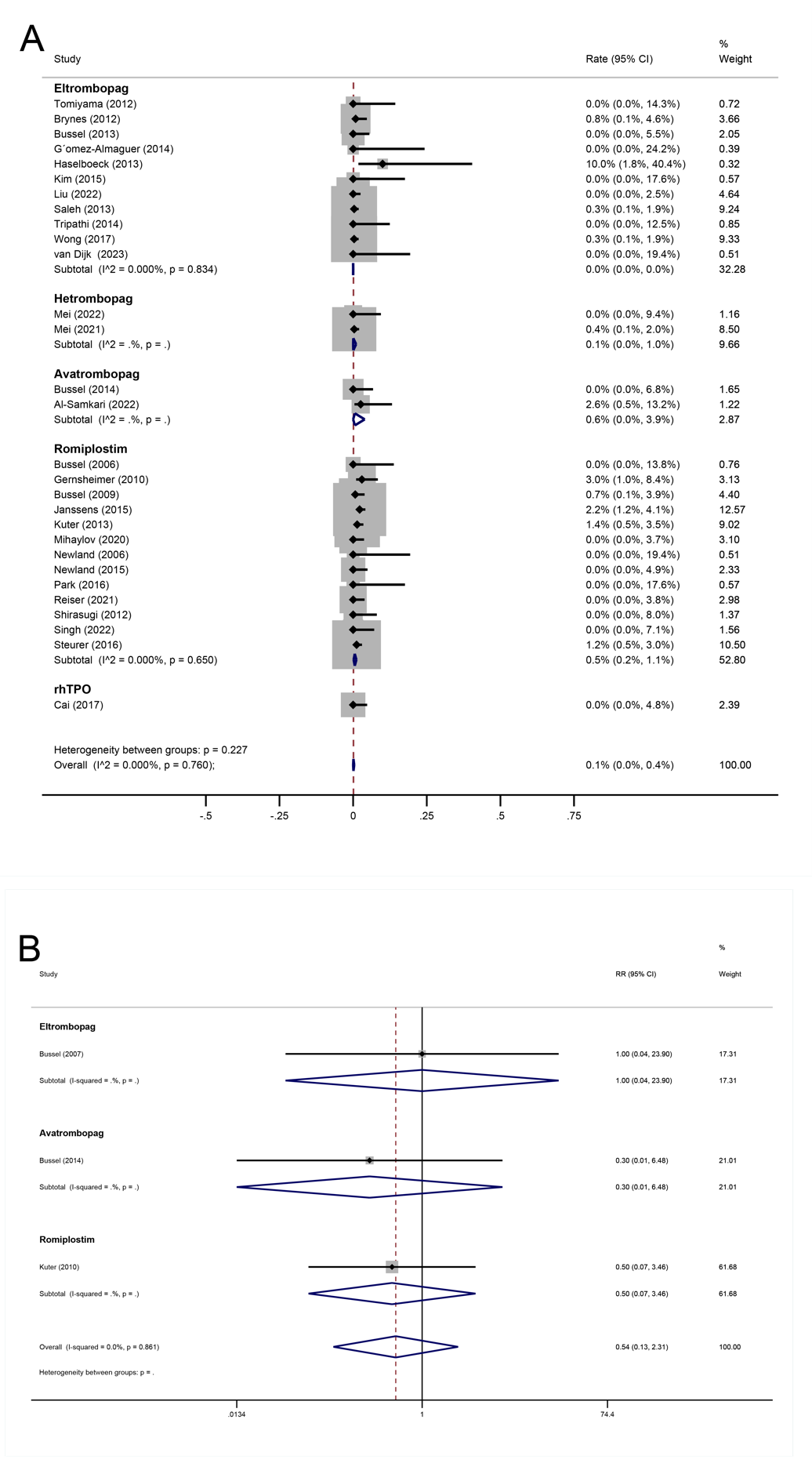


Supplemental Figure 8. Forest plot of other types of venous thrombotic events in ITP patients treated with TAs. (A) Forest plot of rate for other types of venous thrombotic events after ITP patients treated with TAs in single-arm trials. (B) Forest plot of RR for other types of venous thrombotic events after ITP patients treated with TAs in randomized controlled trials. RR: risk ratio. ITP: immune thrombocytopenia. TAs: thrombopoietic agents. CI: confidence interval.


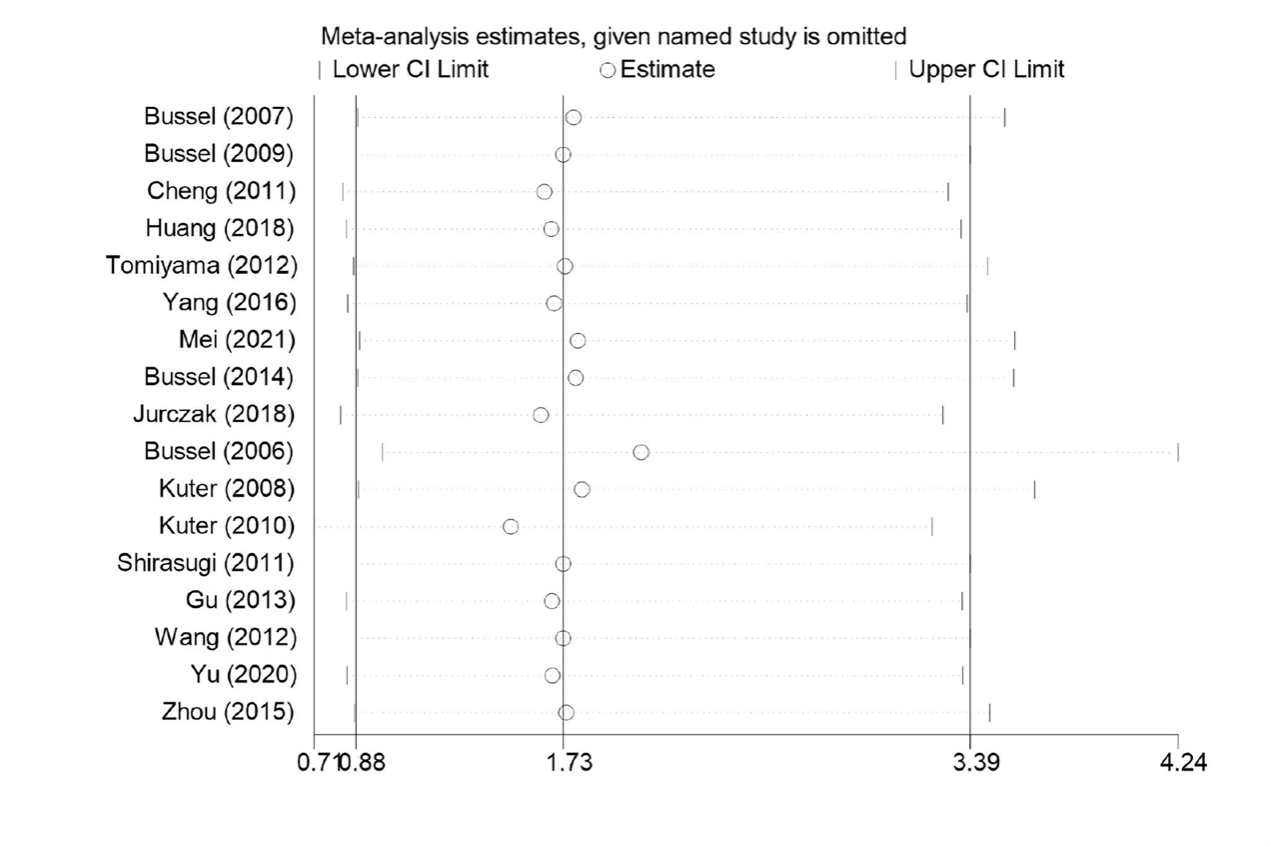


Supplemental Figure 9. Sensitivity analysis for RR of overall thrombotic events performed by using the leave-one-out method. RR: risk ratio.


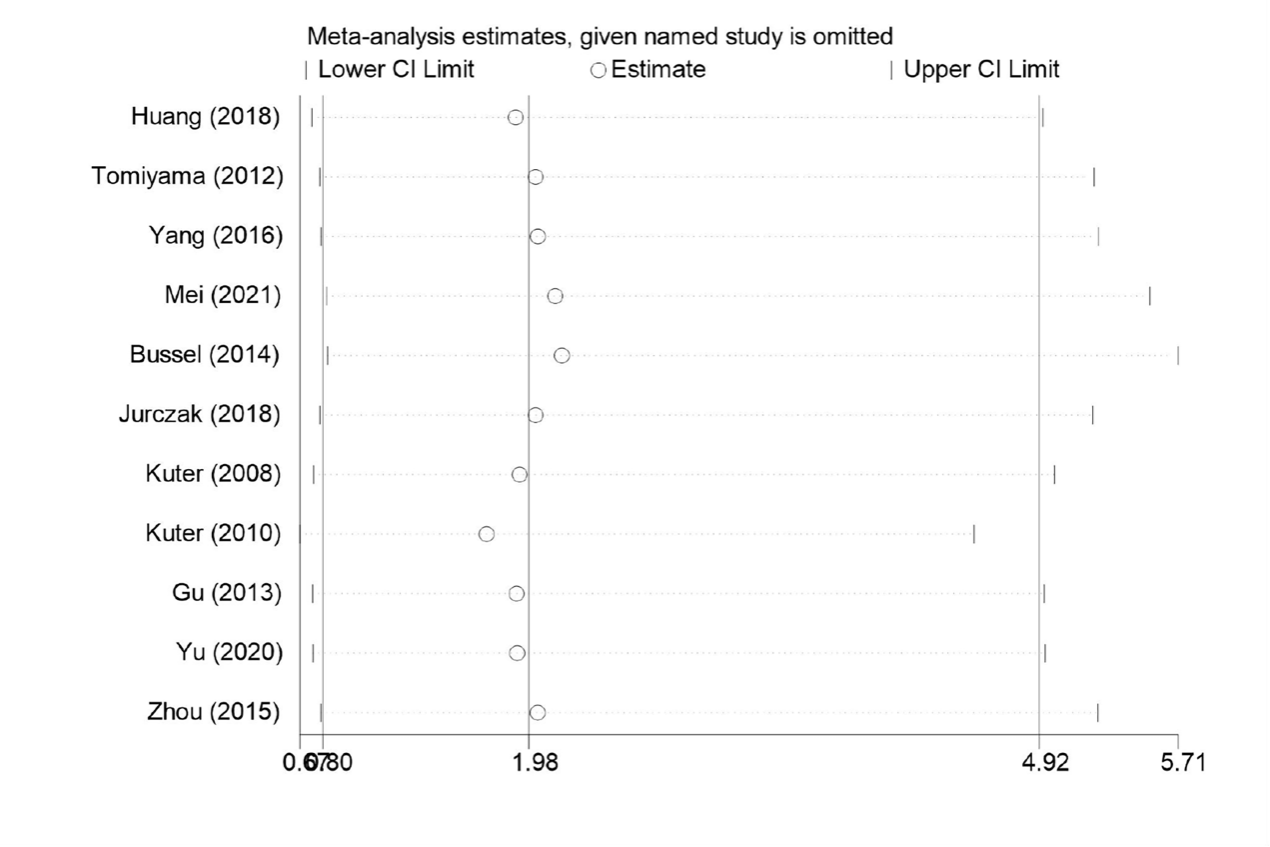


Supplemental Figure 10. Sensitivity analysis for RR of arterial thrombotic events performed by using the leave-one-out method. RR: risk ratio.


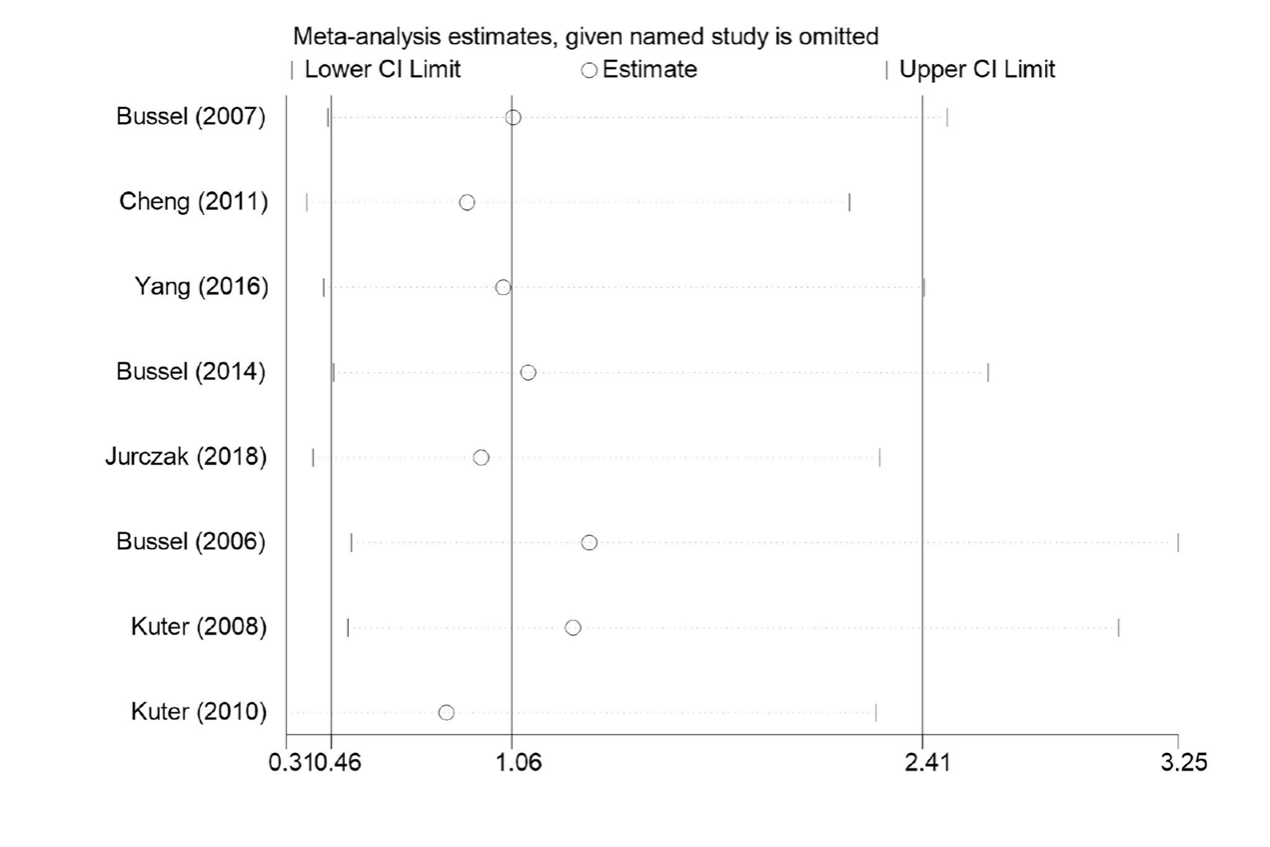


Supplemental Figure 11. Sensitivity analysis for RR of venous thrombotic events performed by using the leave-one-out method. RR: risk ratio.

**
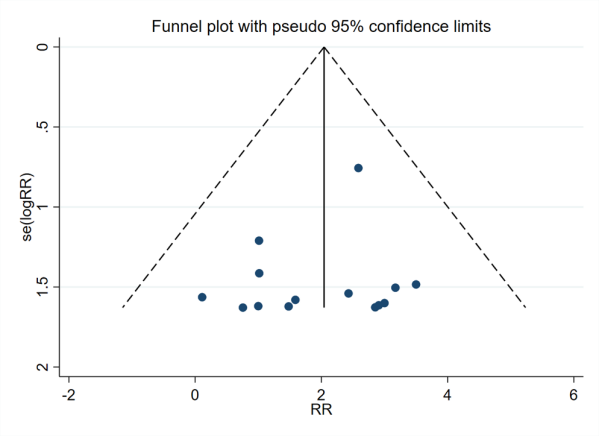
**

Supplemental Figure 12. Funnel plot with pseudo 95% confidence limits of overall thrombotic events.
